# AxoDen: An Algorithm for the Automated Quantification of Axonal Density in Defined Brain Regions

**DOI:** 10.1523/ENEURO.0233-24.2025

**Published:** 2025-06-03

**Authors:** Raquel Adaia Sandoval Ortega, Emmy Li, Oliver Joseph, Pascal A. Dufour, Gregory Corder

**Affiliations:** ^1^Department of Psychiatry, Perelman School of Medicine, University of Pennsylvania, Philadelphia, Pennsylvania; ^2^Department of Neuroscience, Perelman School of Medicine, University of Pennsylvania, Philadelphia, Pennsylvania; ^3^Department of Anesthesiology and Critical Care, Perelman School of Medicine, University of Pennsylvania, Philadelphia, Pennsylvania

**Keywords:** axon, imaging, microscopy, neuron, open-source, toolbox

## Abstract

The rodent brain contains 70,000,000+ neurons interconnected via complex axonal circuits with varying architectures. Neural pathologies are often associated with anatomical changes in these axonal projections and synaptic connections. Notably, axonal density variations of local and long-range projections increase or decrease as a function of the strengthening or weakening, respectively, of the information flow between brain regions. Traditionally, histological quantification of axonal inputs relied on assessing the fluorescence intensity in the brain region of interest. Despite yielding valuable insights, this conventional method is notably susceptible to background fluorescence, postacquisition adjustments, and inter-researcher variability. Additionally, it fails to account for nonuniform innervation across brain regions, thus overlooking critical data such as innervation percentages and axonal distribution patterns. In response to these challenges, we introduce AxoDen, an open-source semiautomated platform designed to increase the speed and rigor of axon quantifications for basic neuroscience discovery. AxoDen processes user-defined brain regions of interests incorporating dynamic thresholding of grayscale-transformed images to facilitate binarized pixel measurements. Here, in mice, we show that AxoDen segregates the image content into signal and nonsignal categories, effectively eliminating background interference and enabling the exclusive measurement of fluorescence from axonal projections. AxoDen provides detailed and accurate representations of axonal density and spatial distribution. AxoDen's advanced yet user-friendly platform enhances the reliability and efficiency of axonal density analysis and facilitates access to unbiased high-quality data analysis with no technical background or coding experience required. AxoDen is *ad libitum* available to everyone as a valuable neuroscience tool for dissecting axonal innervation patterns in precisely defined brain regions.

## Significance Statement

The rodent brain serves as a critical model for understanding brain connectivity and how neural pathologies change the anatomy of neural circuits, which reflect dynamic alterations in information flow. AxoDen, an open-source semiautomated platform, which enhances the speed, accuracy, and rigor of axonal density analysis by employing dynamic thresholding and user-defined regions of interest. The AxoDen tool democratizes access to a high-quality, no-coding-required data analysis pipeline, thereby empowering researchers to unravel the complexities of axonal innervation in precise brain regions, ultimately advancing our understanding of neural circuitry in health and pathology.

## Introduction

Understanding the brain’s structural integrity and connectivity is fundamental in neuroscience (Luo, 2021). Studies ranging from basic anatomy to complex neurological disorders rely on quantification of axonal projections across brain regions to assess changes in information-processing pathways. Accurate measures of axonal density allow to infer potential mechanisms underlying alterations on cognitive, sensory, and motor functions. Consequently, precise axonal quantification is crucial to reveal the structural connectivity in normal and pathological states.

Historically, the mean fluorescence intensity of a sample has been a straightforward method for approximating protein levels. While the complex branching morphology of axons has favored semiquantitative metrics (Fillinger et al., 2018), today’s data-rich environment favors fully quantitative methods. Consequently, intensity-based measurements of brain regions of interest (ROIs) from 2D histological images have dominated in the past years to quantify labeled axonal projections (Beyeler et al., 2016; Powell et al., 2019; Nicolas et al., 2023). While this approach can reveal complex connectivity patterns and pathology effects on neural networks, several limitations—susceptibility to background fluorescence, vulnerability to acquisition settings, and the inherent inter-researcher variability—raise concerns regarding reliability and precision. Furthermore, this approach does not accommodate the innervation heterogeneity within ROIs, neglecting essential information on spatial distribution, and innervation percentage.

Efforts to automate quantification have led to the development of several tools, each with strengths and limitations. MeDUsA (Nitta et al., 2023), created in Python, uses advanced convolutional neural networks (CNNs) to identify an ROI and quantify axon terminals in *Drosophila*’s visual system. MeDUsA achieves high accuracy for *Drosophila*, but its high specificity limits its applicability to other species. An algorithm with higher potential for inter-species utilization is AxonTracer (Patel et al., 2018), created as an ImageJ plugin to measure axonal length in the rat spinal cord. While this tool yields valuable insights into axonal complexity, a skeletonization process that reduces axons to a uniform width of 1 pixel prevents quantification of innervation percentages. The ImageJ macro DEFiNE (Powell et al., 2019) advances the methodology by incorporating an automatic preprocessing stage to diminish background fluorescence before semiautomated quantification. DEFiNE evaluates *Z*-stack images eliminating artifacts and defines signal as pixels exceeding four standard deviations above the mean intensity of axon-free areas. However, DEFiNE requires dual-channel imaging—one for the target fluorophore and another for background fluorescence—limiting its use in experiments using all channels for multiple information levels. Additionally, DEFiNE's need for rectangular images precludes comprehensive brain region innervation analysis. Lastly, TrailMap (Friedmann et al., 2019) transcends the limitations of 2D analysis by employing CNNs to map axonal projections within three-dimensional structures. While CNNs can yield highly accurate data outputs, they can introduce new unknown biases and errors that are difficult to understand due to the black-box behavior of such methods. Indeed, this Python-based algorithm facilitates region-specific quantification of total axonal content in 3D, necessitating intact, lipid-cleared mouse brains imaged with light-sheet microscopy. Despite its precision and analysis depth, TrailMap's reliance on advanced clearing techniques, expensive specialized microscopes, and high computational data processing and storage resources places it beyond the reach of many research laboratories.

These tools exemplify the evolving landscape of neural imaging and analysis, highlighting the ongoing need for methodologies balancing specificity, versatility, and accessibility to accommodate the diverse requirements of neuroscience research. Drawing on established image-processing theory, threshold-based segmentation can reliably separate objects from the background when their intensity distributions are distinct (Cardullo and Hinchcliffe, 2013; Uchida, 2013). Although widely used for tasks like cell and colony counting, this approach remains underutilized for axonal quantification due to the complex, branching nature of axons. Here, we introduce a simple, open-source, and reproducible segmentation-based algorithm for axonal quantification—AxoDen.

## Materials and Methods

### Animals

All experimental procedures were approved by the Institutional Animal Care and Use Committee of the University of Pennsylvania and performed in accordance with the US National Institutes of Health guidelines. Mice aged 2–5 months were housed 2–5 per cage and maintained on a 12 h reverse light/dark cycle in a temperature and humidity-controlled environment with *ad libitum* food and water.

Mice genetic background was the following for each set of images:

1. Red-fluorescently labeled axons of the images used for the validation of AxoDen were from TRAP2 mice crossed with CAG Sun1 [B6;129-Gt(ROSA)26Sortm5(CAG-Sun1/sfGFP)Nat/J] reporter mice that express a GFP fluorophore in a Cre-dependent manner (“TRAP2:CAG-Sun1 sfGFP”). These were purchased from Jackson Laboratory, strain #021039, and bred to homozygosity for both genes. This group consisted of three males and one female.
2. Images of green-fluorescently labeled axons were from TRAP2 mice crossed with Ai9 [B6.Cg-Gt(ROSA)26Sortm9(CAG-tdTomato)Hze/J] reporter mice expressing a tdTomato fluorophore in a Cre-dependent manner (“TRAP2:tdTomato”). Purchased from Jackson Laboratory, strain #007909 and bred to homozygosity for both genes. This animal was a male.
3. C57BL/6J wild-type female mice were used for the image of axons innervating the anterior cingulate cortex (ACC) and were purchased from Jackson Laboratory, strain #000664.

### Viral vectors

All viral vectors were either purchased from Addgene.org, or custom designed and packaged by the authors as indicated. All AAVs were aliquoted and stored at −80°C until use and then stored at 4°C for a maximum of 4 d. The next two AAVs were used for the red-fluorescently labeled axons of the images used for the validation of AxoDen:

1. AAV5-*mMORp*-FlpO (Stanford Viral Vector Core; titer, 1.9 × 10^12^ vg/ml; volume, 100 nl)
2. AAV8-*Ef1a*-Con/Fon-oScarlet (Addgene 137136-AAV8; titer, 2.2 × 10^12^ vg/ml; volume, 100 nl)

The following AAV was used for the green-fluorescently labeled axons:

- AAV5-*hSyn*-DIO-EGFP (Addgene 50457-AAV5; titer: 1.3 × 10^12^ vg/ml, volume: 400 nl)

For the image of axons innervating the ACC, the following AAV was used:

- AAV1-*mMORp*-hM4Di-mCherry (Stanford Viral Vector Core; titer, 1.17 × 10^12^ vg/ml; volume, 400 nl)

### Stereotaxic surgery

Adult mice (∼8–10 weeks of age) were anesthetized with isoflurane gas in oxygen (initial dose, 5%; maintenance dose, 1.5%) and fitted into Kopf stereotaxic frames for all surgical procedures. The 10 µl Nanofil Hamilton syringes (WPI) with 33 G beveled needles were used to intracranially infuse AAVs into the different brain areas of interest. Based on the Paxinos mouse brain atlas, the following coordinates, relative from the bregma, were used for each set of images:

1. Images of red-fluorescently labeled axons used for the validation of AxoDen: ACC, AP, −1.50 mm; ML, ±0.3 mm; DV, −1.5 mm
2. Images of green-fluorescently labeled axons: basolateral amygdala (BLA), AP, −1.20 mm; ML, 3.20 mm; DV, −5.20 mm
3. Image of axons innervating the ACC, the following AAVs were used: centromedial thalamic nucleus (CM), AP, −1.70 mm; ML, 0.70 mm; DV, −4.00 mm; angle, 10°.

Mice were given a 3–8 week recovery period to allow ample time for viral diffusion and transduction to occur. For all surgical procedures, meloxicam (5 mg/kg) was administered subcutaneously at the start of the surgery, and a single 0.25 ml injection of sterile saline was provided upon completion. All mice were monitored and given meloxicam for up to 3 d following surgical procedures.

### TRAP protocol (tamoxifen induction)

The images of red-fluorescently labeled axons used for the validation of AxoDen were from pain-active neurons in the ACC (James et al., 2024), labeled via tamoxifen induction. Mice were habituated to the testing room the day before TRAP execution, and no nociceptive stimuli were delivered. On both days of habituation and TRAP procedure, mice were placed within red plastic cylinders (10.16 cm in diameter), with a red lid, in a raised metal grid. On the day of the TRAP procedure, mice were habituated to the room and the cylinder for 60 min, and then they received 20 stimuli consisting of a water drop at 55°C interspaced by 30 s over 10 min. Following the stimulation, the mice remained in the cylinder for an additional 60 min before injection of 4-hydroxytamoxifen (20 mg/kg in ∼0.25 ml vehicle, s.c.). After the injection, mice remained in the cylinder for an additional 2 h to match the temporal profile for c-Fos expression, at which time the mice were returned to the home cage.

### Tissue processing

Animals were anesthetized using FatalPlus (Vortech, 100 µl) and transcardially perfused with 0.1 M phosphate buffered saline (PBS), followed by 10% normal buffered formalin (NBF) solution (Sigma-Aldrich, HT501128). Brains were quickly removed and postfixed in 10% NBF for 24 h at 4°C and then cryo-protected in a 30% sucrose solution made in 0.1 M PBS until sinking to the bottom of their storage tube (∼48 h). Brains were then frozen in Tissue-Tek O.C.T. compound (Thermo Fisher Scientific) and coronally sectioned on a cryostat (CM3050S, Leica Biosystems) at 30 μm, and the sections were stored in 0.1 M PBS. TRAPped animals were perfused 4 weeks after the TRAP protocol.

### Fluorophore amplification

Floating sections were permeabilized in a solution of 0.1 M PBS containing 0.3% Triton X-100 (PBS-T) for 30 min at room temperature and then blocked in a solution of 0.3% PBS-T and 5% normal donkey serum (NDS) for 2 h before being incubated with primary antibodies in a 0.3% PBS-T, 5% NDS solution for ∼16 h at room temperature. Following washing three times for 10 min in PBS-T, secondary antibodies prepared in a 0.3% PBS-T, 5% NDS solution were applied for ∼2 h at room temperature, after which the sections were washed again three times for 5 mins in PBS-T, then again three times for 10 min in PBS-T, and then counterstained in a solution of 0.1 M PBS containing DAPI (1:10,000, Sigma-Aldrich, D9542). Fully stained sections were mounted onto Superfrost Plus microscope slides (Thermo Fisher Scientific) and allowed to dry and adhere to the slides before being mounted with Fluoromount-G Mounting Medium (Invitrogen, 00-4958-02) and coverslipped.

For each batch of images, the antibodies included:

1. Images of red-fluorescently labeled axons of the images used for the validation of AxoDen:
  a. 1°Ab: rabbit anti-dsRed [1:1,000, Takara, 632496]
  b. 2°Ab: Alexa Fluor 555 donkey anti-rabbit [1:500, Invitrogen Thermo Fisher Scientific, A31572]
2. Images of green-fluorescently labeled axons:
  a. 1°Ab: chicken anti-GFP [1:1,000, Abcam, ab13970]
  b. 2°Ab: Alexa Fluor 488 donkey anti-chicken [1:500, Jackson ImmunoResearch Laboratories, 703-545-155]
3. Image of red-fluorescently labeled axons innervating the ACC:
  a. 1°Ab: rabbit anti-dsRed [1:1,000, Takara Bio, 632496]
  b. 2°Ab: Alexa Fluor 594 donkey anti-rabbit [1:500, Abcam, A21207]

### Imaging

All images were acquired with the 20× objective Nikon PlanApo 20× 0.75NA/0.60 mm working distance, using the Keyence microscope BZ-X810.

### Datasets

All images were generated in the Corder Lab as described in the methods above.

### Axonal quantification using mean fluorescence intensity

All images were initially acquired and stored in their original format without any brightness or contrast modifications. When opening the images in FIJI for ROI selection, we temporarily adjusted brightness and contrast to aid visualization of axons within the ROI. This step did not alter the underlying pixel values. After identifying and outlining the ROI (using a rectangular selection tool that remained entirely within the boundaries of the anatomical region), we reverted the display settings to the original, unmodified data. We then used the “Histogram” function in FIJI’s “Analyze” menu to measure the mean pixel value within the selected ROI. This approach ensures that any brightness or contrast changes for visualization do not affect the actual intensity measurements, which are taken directly from the unaltered data. Data on the mean intensity fluorescence were manually typed in Excel files.

### Statistics

**Figure 1.**
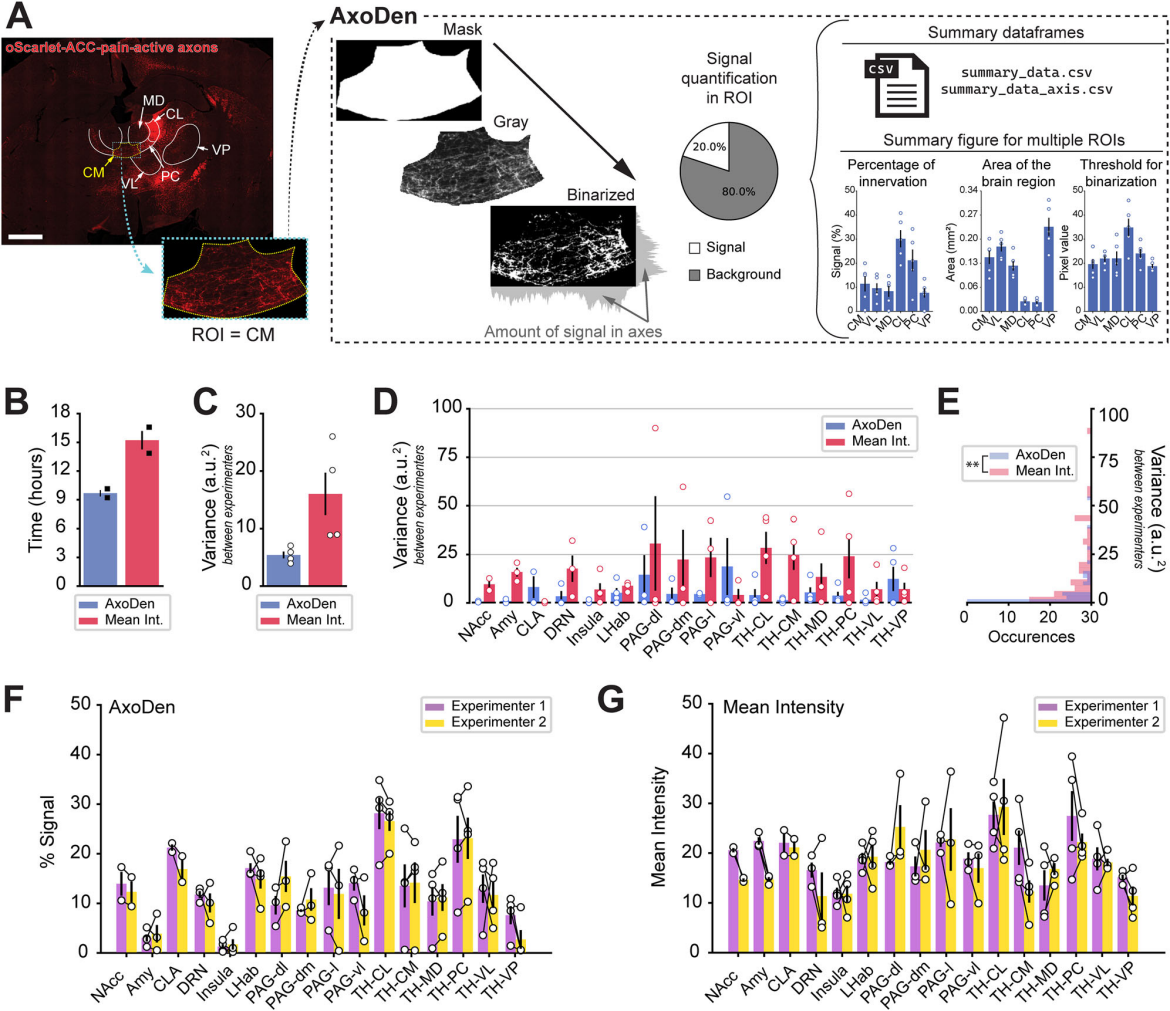
AxoDen overview and comparison to mean intensity method. ***A***, Overview of AxoDen steps and outcome using example oScarlet-ACC-pain-active axons projecting to midline thalamic nuclei. Scale bar, 1 mm. ***B***, Time needed to collect the data for AxoDen and mean intensity. Each black square represents one experimenter. ***C***, Overall variance between researchers for AxoDen and mean intensity. ***D***, Variance between experimenters when using AxoDen and mean intensity for multiple brain regions. ***E***, Distribution of the variance between researchers for each method. Mann–Whitney *U* test revealed AxoDen has an overall lower variance than mean intensity. **p < 0.001. ***F***, Percentage of signal in different ROIs indicating the percentage of the ROI receiving axonal projections for each experimenter using AxoDen. ***G***, Mean fluorescence intensity values collected for each brain area by different experimenters. ***A***, ***C*–*G*** Each dot represents one animal. All values are mean ± SEM. ACC, anterior cingulate cortex; Amy, amygdala; CL, centrolateral nucleus; CLA, claustrum; CM, centromedial nucleus; dl, dorsolateral; dm, dorsomedial; DRN, dorsal raphe nucleus; l, lateral; LHab, lateral habenula; MD, mediodorsal nucleus; NAcc, nucleus accumbens; PAG, periaqueductal gray; PC, paracentral nucleus; PV, paraventricular nucleus; ROI, region of interest; TH, thalamus; vl, ventrolateral; VL, ventrolateral nucleus; VP, ventroposterior nucleus.

The variance between researchers of Figure 1*C*–*E* was calculated as follows: First, for each brain region of each animal, the value obtained by Researcher A and that of Researcher B were collected. Then, the variance was calculated with the Python function *var* from the *numpy* library. Last, the variance of all brain regions of one animal were averaged so that each animal is one circle in Figure 1, *C* and *D*. To run the statistics of the variance in Figure 1*E*, given the skewed distribution of the dataset, we used the nonparametric Mann–Whitney *U* test, using the Python function *stats.mannwhitneyu* from the *scipy* library.

### Code accessibility

To improve reproducibility between labs, we have made AxoDen available for everyone, independently of the technical capabilities and the level of coding expertise. The source code is available at https://github.com/raqueladaia/AxoDen. However, for reproducibility, the source code must be accompanied by configuration files (i.e., environments, dependencies, and parameters). Therefore, to make it more accessible and reproducible for users without coding experience, AxoDen is available as a Graphical User Interface (GUI) that can be downloaded at https://github.com/raqueladaia/AxoDen/releases for Windows, MacOS, and Linux/Ubuntu users. Standard desktops and laptops have the capability to run AxoDen. To further reduce the barrier of entry, we also provide AxoDen as a web application at https://axoden.streamlit.app/, which can be accessed through a browser. Example images to test AxoDen can be downloaded at the https://github.com/raqueladaia/AxoDen. Tutorials can be found at the GitHub repository and at the web application. Additional information can be found in www.corderlab.com and https://sandovalortega.com/.

## Results

### AxoDen: a streamlined pipeline for axonal density quantification

Recognizing the challenges of currently available analysis pipeline for axonal quantification in 2D brain samples, we have built AxoDen (Fig. 1*A*)—a versatile histology image analysis platform that refines and simplifies the process of quantifying axonal inputs, is applicable to any animal species and fluorophores, does not require an advanced setup nor GPU, and uses only one channel or fluorophore. Beyond the traditional reliance on measurement areas that do not follow the anatomical contour of a brain ROI, AxoDen processes images that have been masked and cropped to specifically fit the brain ROIs, conforming to the actual contours identified in the brain atlas selected by the user. This approach allows for the isolation and exclusive measurement of fluorescence signals from axonal projections within the defined brain region.

By incorporating the Otsu dynamic thresholding method for the binarization of grayscale images (Otsu, 1979), our protocol distinguishes between signal and nonsignal elements within the brain region, effectively minimizing background interference and consequently ensuring data collection focuses solely on axonal information. The quantification of fluorescently labeled axons is performed for the whole ROI and is also projected to both the *x* and the *y* axis for the spatial analysis of projections. The data on the signal percentage, ROIs area, and threshold used for binarization are saved as data frames in CSV files that the user can retrieve for later statistical evaluation on their preferred analysis software (GraphPad Prism, ggplot2, R, MATLAB or Python). Furthermore, AxoDen generates a summary figure containing information on these parameters for the immediate visualization of the results (as mean ± SEM) after running the analysis (Fig. 1*A*). The panel showing the signal percentage for each brain region contains the results of the analysis. The panels of the ROI areas, and the pixel values used for binarizing the images are important to confirm the quality of the dataset (see subsection “Output 1: Summary data plo” within section “Interpretation of the figures created by AxoDen” for detailed description).

To validate AxoDen and compare it to the intensity-based methods measuring fluorescence intensity, we used a dataset of 46 images of pain-active axons originating from the ACC (James et al., 2024). Two experimenters selected 19 brain ROIs from four mice, and each of them generated a new dataset consisting of 76 items. We first compared the time needed by each researcher to prepare the images for AxoDen analysis with the time required with the intensity-based method, for which we chose a rectangular shape to follow the same approach as recently published papers (Beyeler et al., 2016; Nicolas et al., 2023). We refer the later as mean intensity from now on. Preparing the data for AxoDen was 1.6 times faster than collecting the data for mean intensity analysis (AxoDen, 9.68 ± 0.33 h; mean intensity, 15.22 ± 0.96 h; Fig. 1*B*).

We additionally evaluated the variance between researchers for each brain area and mouse (Fig. 1*C*), excluding images with poor background fluorescence for which AxoDen was not able to distinguish “signal” from “background.” Among the four mice analyzed, AxoDen showed a lower mean inter-researcher variability compared with mean intensity analysis (AxoDen, 5.42 ± 0.58; mean intensity, 16.01 ± 3.69). This effect was observed in 13 of the 16 included ROIs (Fig. 1*D*). Because the data were highly skewed, we used a Mann–Whitney test (*p* = 0.0002) to compare the two methods (Fig. 1*E*). The results were further supported by direct comparisons of the measurements taken by each experimenter, which revealed that AxoDen produced more consistent values (Fig. 1*F*) than mean intensity analysis (Fig. 1*G*). Altogether, these findings demonstrate that (1) AxoDen reduces the time required for data collection and analysis and (2) AxoDen lowers interexperimenter variability.

### Experiment overview for axonal quantification using AxoDen

**Figure 2.**
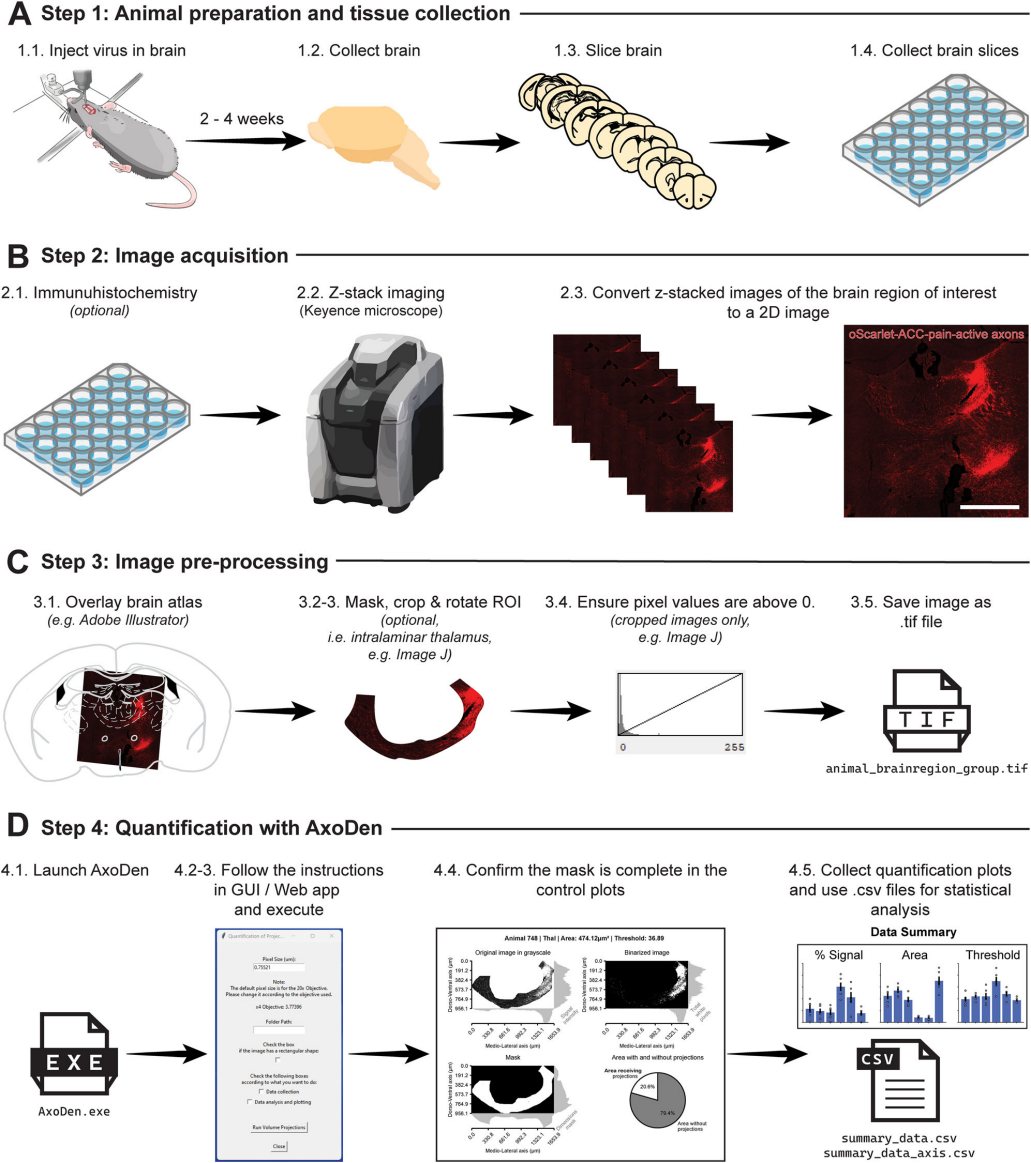
Experimental workflow for anatomical analysis of axonal projections in the intralaminar thalamus. ***A***, Steps for animal preparation and tissue collection. ***B***, Steps for image acquisition. Scale bar, 1 mm. ***C***, Steps for image preprocessing. ***D***, Steps for axonal quantification with AxoDen and its outputs.

AxoDen has been designed to provide a streamlined analysis of axonal projections in anatomical studies. Therefore, the workflow for quantifying axonal innervations of predefined brain ROIs follows a four-step process (Fig. 2):

**Step 1: Animal preparation and tissue collection** (Fig. 2*A*)

1. Viral Vector Injection: Administer a viral vector containing a fluorescent reporter into the targeted brain region of the animal model of choice to label axonal projections.
2. Expression Period: Allow a period of 2–4 weeks for optimal viral expression in neuronal projections.
3. Euthanasia and Brain Harvesting: Euthanize the animal following approved ethical guidelines and carefully extract the brain.
4. Tissue Sectioning: Slice the brain into sections ranging from 30 to 50 μm in thickness, suitable for subsequent imaging.

**Step 2: Image acquisition** (Fig. 2*B*)

1. Immunohistochemistry (Optional): If necessary, apply immunohistochemistry techniques to amplify the fluorescent signal to ensure visibility—or increase the signal-to-noise ratio (SNR)—of axonal projections.
2. *Z*-Stack Imaging: Capture the axonal projections at multiple depths of the brain ROI by acquiring *Z*-stacks using a 20× objective lens on a microscope.
3. Image Processing: Process the *Z*-stacks using a maximal projection mode to generate 2D images that minimize background fluorescence while preserving signal integrity.

**Step 3: Image preprocessing** (Fig. 2*C*)

1. Atlas Overlay: Superimpose the relevant brain atlas onto your images to accurately identify the ROIs for the later masking step.
2. Region Masking: Precisely delineate, mask, and crop the brain area of interest, guided by the overlay created previously to ensure accurate measurement zones. We recommend this step to be performed in software such as ImageJ.
3. Image Rotation (Optional): Rotate the image to align the mediodorsal axis to the *x* axis and the dorsoventral axis to the *y* axis. This step can allow for a detailed study of differential innervation of cortical layers. For details read the section Consideration 3.
4. For cropped and masked images only: Ensure there are no black pixels in the tissue, as these are considered void of information. For details read the sections Considerations 1, 2, and 3.
5. Image Batch Naming: Save all processed and cropped images in the format animalID_brainregion_group_[additional_information].tif to follow the data management organization of the algorithm for batch processing. For details read the section Consideration 5.

**Step 4: Quantification with AxoDen** (Fig. 2*D*)

1. Script Initialization: Launch AxoDen in any of its available forms.
2. Provide the requested information to the GUI:
  - Pixel Size: The default pixel size is for a 20× objective [Nikon PlanApo 20× 0.75NA/0.60 mm working distance] in a Keyence microscope [BZ-X Series].
  - Input the data:
    - Users of the web app upload the images to analyze.
    - Users of the executable provide the directory path containing the saved images to the script interface.
    - Users of the Python package or the cloned repository can use either of the methods to input their data.
    Toggle option if the images are masked and cropped to a defined region of interest.
3. Analysis Execution: Run the script to initiate automated axonal quantification. In the stand-alone GUI, the control plots, summary figures, and .CSV files containing the data frames generated during the analysis will be saved in the provided output folder. In the Web application, the user can decide which information to download.
4. Check the control plots: Make sure the mask covers the entire area of the brain ROI.
5. Collect the data of interest from the output files:
  - CSV files contain the raw data used to create the figures and can be used by the user for later statistical analysis:
    - One CSV file provides information on the overall innervation of each brain area and animal.
    - The second CSV file provides information on the fluorescence intensity along the *x* and the *y* axis of the images.
  - PDF files contain figures that the user can open and modify in Adobe Illustrator.

### Interpretation of the figures created by AxoDen

AxoDen outputs three different types of plots.

#### Output 1: summary data plot

The summary plot consists of three panels of bar graphs where the bar is the mean ± SEM and the dots are animals, provided the user has following the naming convention of AxoDen. The three panels from left to right are:

Panel 1. Overall innervation density represented as the percentage of signal measured in the ROI. The percentage of signal is the percentage of white pixels.

Panel 2. The area of each ROI. When the user provides the pixel size in micrometer, the resulting area will be expressed in square micrometers (µm^2^). It is important to ensure that the data points are appropriately clustered. If an outlier is detected—a data point with a value significantly different from the others—the control plots for the corresponding brain region should be revisited to investigate the cause. This anomaly may indicate issues in the cropping step of the image.

Panel 3. Pixel value used as threshold for the binarization step. Pixel values should exhibit a sufficient degree of clustering to ensure reliable analysis. If the values display large variance or if an individual value significantly deviates from the rest, it is important to examine the control plots to identify the cause of these discrepancies and correct as necessary.

#### Output 2: summary data axis plot

These plots show, for each brain region, the distribution of the signal across the *x* and the *y* axis. The gray lines represent animals, and the blue line with the shade surrounding it the mean ± SEM.

#### Output 3: control plots

AxoDen generates one control plot for each image it processes. Each control plot provides the following information:

Top left. Original image in gray scale with the amount of signal in the *x* and *y* axis.

Bottom left. Mask. In masked and cropped ROIs, it is essential to ensure that the mask fully covers the ROI. A patchy or incomplete mask indicates that areas of background fluorescence have zero values, which are interpreted as missing data. This issue can lead to an overestimation of the signal-to-background ratio and result in misleading outcomes. The dimensions of the mask are plotted along the *x* and *y* axes. For rectangular images, the mask does not provide relevant information.

Top right. Binarized image. After applying Otsu dynamic thresholding, the original image is converted into a binarized black-and-white version. It is essential for the user to ensure that all axons visible in the original image are accurately represented in the binarized image. If axons appear overrepresented or underrepresented, the user should consider reacquiring the image or adjusting the pixel intensity range. Additionally, the amount of signal along the axes is computed as the sum of white pixels in each row or column along the *x* and *y* axes, respectively, for optional spatial analysis.

Bottom right. Quantification. Pie plot indicating the overall percent of ROI receiving projections.

### AxoDen algorithm workflow

**Figure 3.**
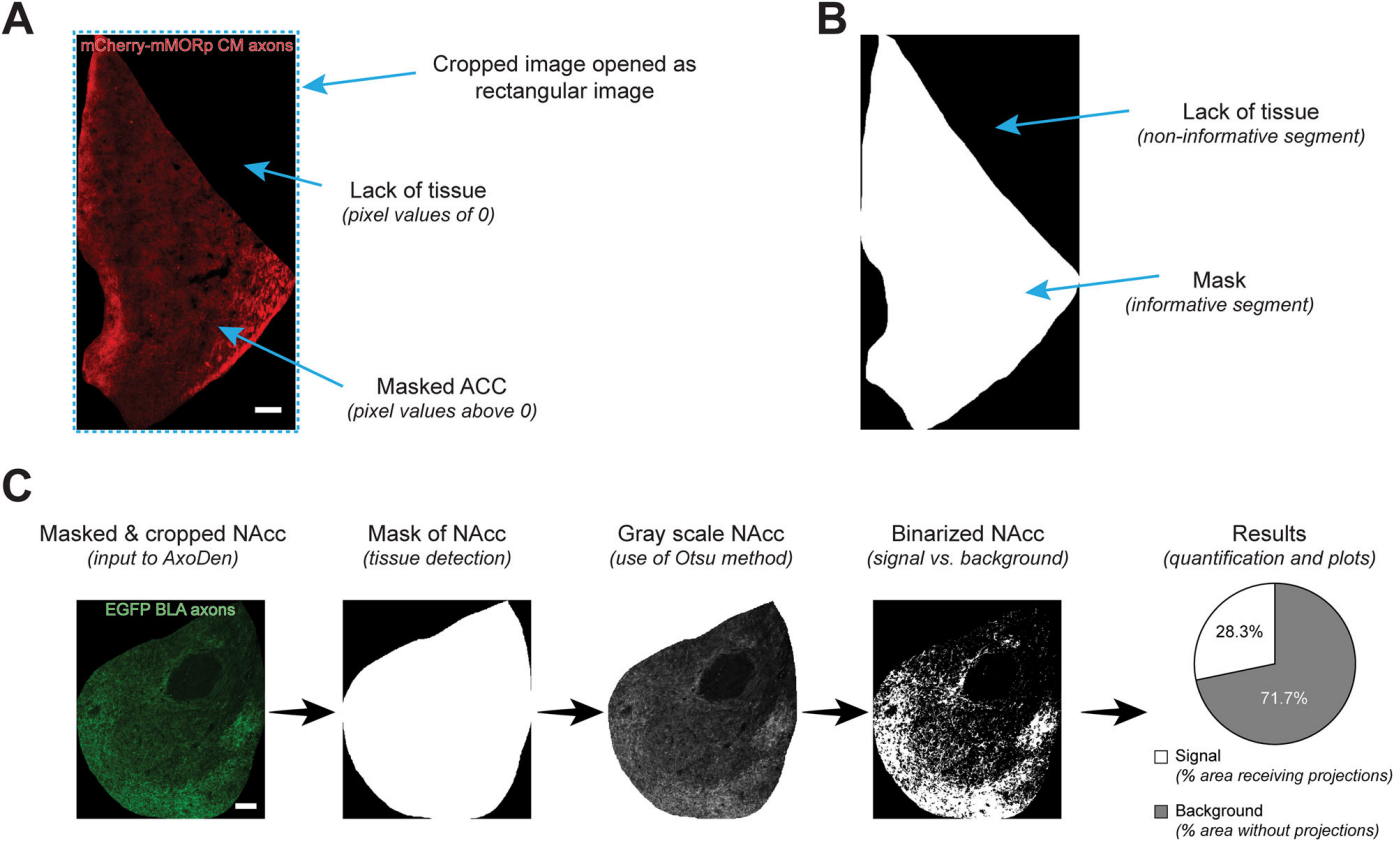
AxoDen workflow with masked and cropped images. ***A***, Components of a masked and cropped image of the ACC receiving CM axons positive for mu opioid receptors labeled with mCherry. ***B***, Illustration of the detected mask by AxoDen from the masked and cropped ACC image. ***C***, AxoDen workflow for an image of the masked and cropped nucleus accumbens (NAcc) with projections labeled with GFP. All scale bars, 100 µm.

The AxoDen algorithm processes images that have been previously masked and cropped to specific shapes, as well as unmasked images. Masked and cropped images are conventionally treated as rectangular frames, with areas lacking tissue represented by black pixels or a value of zero (Fig. 3*A*). This format is used to retrieve the mask, which delineates the boundaries of the chosen brain region, thereby differentiating between informative (tissue-containing) and noninformative (masked-out) segments of the image (Fig. 3*B*). For this reason, it is important that the tissue area does not contain pixels with values of zero (see Consideration 3).

This analysis is independent of the fluorophore used to label projections, given that AxoDen converts all input images into gray scale (Fig. 3*C*). After images have been transformed to gray scale, the Otsu method (Otsu, 1979) is applied using the filters.threshold_otsu function from the scikit-image Python library, which calculates an optimal threshold by finding a value that maximizes the variance between classes. In our case, classes are “signal” and “background.” The resulting threshold is applied to binarize the grayscale image such that pixels above the threshold are classified as signal and given a value of 1 and pixels below the threshold are considered background and are assigned a value of 0, ensuring signal is segregated from background. Taking the binarized image, AxoDen calculates the percentage of the brain region identified with the mask that corresponds to signal versus background, thereby offering an overall view of the percent of innervation of the ROI (Fig. 3*C*). It is important to note that the Otsu dynamic thresholding process is applied regardless of whether axons are actually present in the image. As a result, a bright image containing no axons may still be erroneously segmented, consequently yielding a high percentage of innervation. In such cases, it is crucial for users to verify the true absence of axons by reviewing the provided control plots.

AxoDen additionally offers the possibility to collect the signal levels longitudinally (*x* axis) and latitudinally (*y* axis), offering insights into the innervation across different anatomical axes when the brain region is appropriately aligned with the mediolateral and ventrodorsal axes. Read Consideration 4 for this optional feature.

### Availability

Scientific research has long faced a reproducibility crisis, primarily due to the challenges in replicating experimental conditions and analyses across different laboratories. To address this issue, it is crucial to develop standardized procedures and analyses that are user-friendly for the entire scientific community. AxoDen has been designed with enhanced usability and accessibility in mind, standardizing the quantification of axonal projections. As a result, AxoDen is *ad libitum* available to all scientists, regardless of their familiarity with analysis scripts or coding languages.

AxoDen can be used in three distinct modes, ranked here from requiring the least coding expertise to the developer level, for those who need to modify or integrate AxoDen's functions into their own scripts:

**1. Web Application.** AxoDen is accessible as a web application to anyone through a web browser without signup or user account required. This offers a platform-independent solution that performs the computations in the cloud, broadening AxoDen usability. Any image uploaded here is not saved in any web server and remains in memory only for the analysis, ensuring the confidentiality of any sort of data the user analyzes. The web application can be found here https://axoden.streamlit.app/.
**2. Executable.** AxoDen is available as a stand-alone GUI for Windows, MacOS and Ubuntu/Linux. These stand-alone AxoDen GUI versions can be downloaded here https://github.com/raqueladaia/AxoDen/releases as zip files. After extracting the content of the zip files, the executable will be ready to launch by double clicking on it. The messages that appear in the stand-alone GUI inform about the steps the algorithm is performing.
**3. Source Code.** For those researchers who wish to inspect the source code, the underlying scripts of AxoDen can be accessed by either (1) cloning of GitHub Repository (https://github.com/raqueladaia/AxoDen) or (2) installing the Python pip package axoden (https://pypi.org/project/axoden/). This flexibility allows for tailored modifications of the algorithm to suit specific research needs.

Each function of AxoDen is documented in the documentation website of AxoDen: https://raqueladaia.github.io/AxoDen/. The instructions on how to use, install, or download AxoDen can be found in the GitHub repository. In the Web App application, a “How To” section provides instructions on how to prepare for and use AxoDen on the web browser. Using AxoDen via cloning of the repository or the pip installation package gives access to both the GUI and the local use of the web application. These diverse formats enhance AxoDen's accessibility and adaptability, catering to varying user preferences and technical infrastructures.

### Considerations for proper use of AxoDen

Every digital image is a mosaic of pixels, each encoding information about color and intensity. Commonly, cameras produce color images by synthesizing three primary colors—referred to as channels: red, green, and blue (RGB). Hence, an image inherently possesses three dimensions: (1) width (*x* axis), (2) height (*y* axis), and (3) the color channels (*z* axis). This structure allows us to conceptualize an image as a three-dimensional matrix, wherein each pixel is represented by a trio of values that correspond to the intensity levels of RGB. The linear combination of the intensity values of the various channels give rise to the wide spectrum of colors we observe in an image. Typically, images are captured in an 8 bit format, meaning the pixel intensity in each channel can assume 2^8^, or 256, possible values ranging from 0 to 255 in steps of 1. In this format, a value of 0 across all channels results in black, while a value of 255 across all channels manifests as white. In images acquired at larger resolution (i.e., 16 bit), the image software will likely show the pixel range between 0 and 255. However, given the increased resolution, the steps between values are smaller than 1.

With these fundamentals in place, adhesion to the first three **considerations ensures precise axonal detection.**

### Consideration 1: large brightness differences in adjacent brain regions may require postacquisition adjustments of ROIs with low SNR

**Figure 4.**
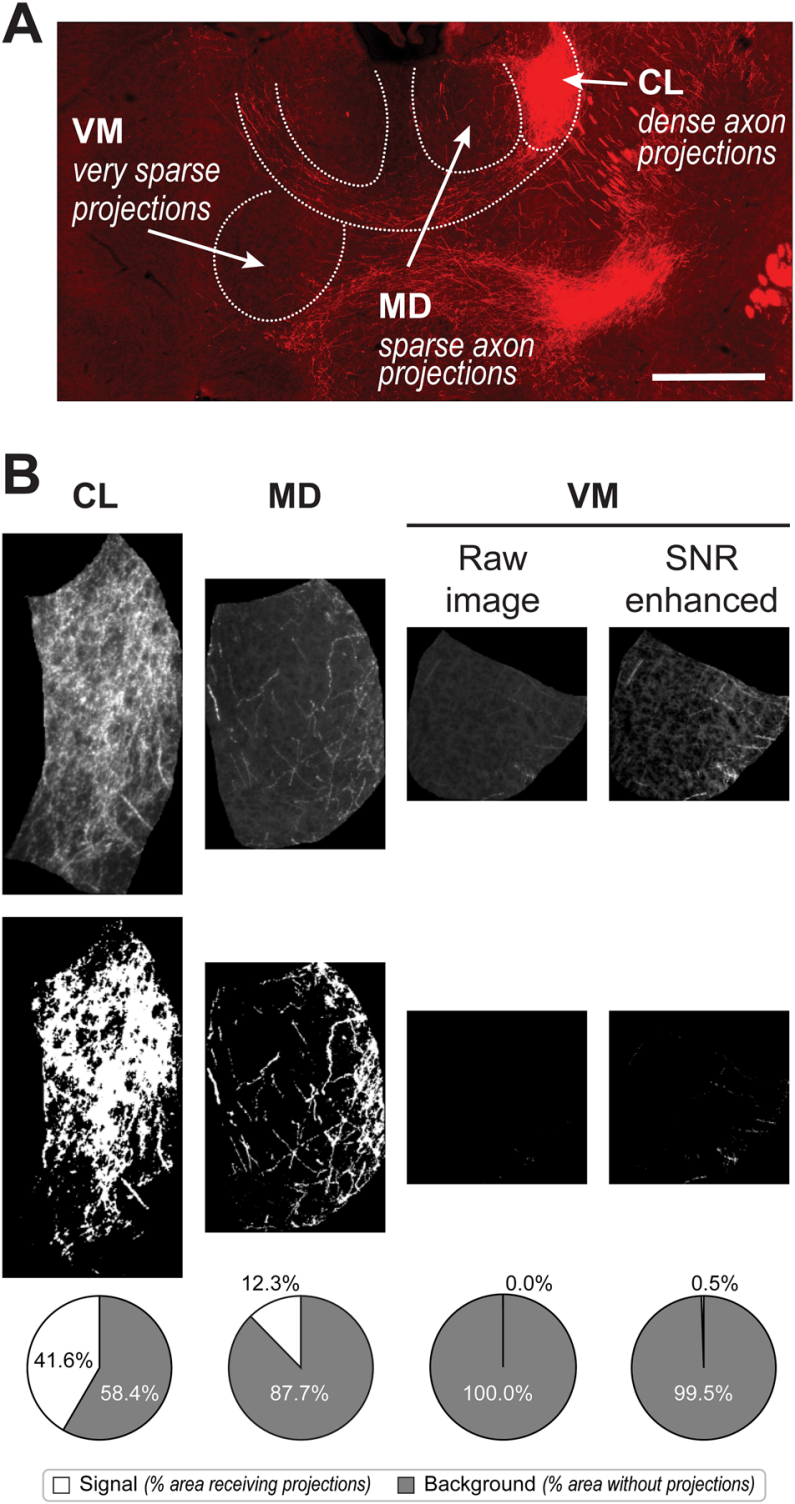
Adjustment of pixel range. ***A***, Example image of oScarlet-labeled projections from the ACC to the thalamus. Three thalamic nuclei receiving strong, weak, and very weak projections are identified. The intensity of the image has been increased for visualization purposes. Scale bar, 1 mm. ***B***, Cropped brain regions in gray scale (top row), after binarization (middle row) and the quantification of signal versus background by AxoDen (bottom row).

Adjacent brain regions may exhibit significantly different levels of innervation (Fig. 4*A*). Consequently, during image acquisition, exposure settings are typically optimized for regions with higher fluorescence to prevent irreversible fluorophore bleaching. This approach ensures that areas with sufficient projections have an adequate dynamic range for AxoDen to effectively distinguish signal from background noise (Fig. 4*B*, CL and MD). However, regions with fewer projections present a reduced dynamic range and poor SNR (Fig. 4*B*, VM raw image). Without postacquisition adjustments after cropping the images, the signal intensity of axons projecting to the VM region remains too low for AxoDen to detect (Fig. 4*B*, VM raw image). One solution to avoid reimaging at higher exposure times is to adjust the pixel intensity range of the ROI with low SNR. The resulting improvement in SNR can enable AxoDen to accurately segment “signal” from “nonsignal” pixels, successfully detecting the axons in the VM region (Fig. 4*B*, VM SNR enhanced). It is essential the user does not modify the pixel range when measuring fluorescence intensity via the traditional positioning of rectangles or other ROIs as it can introduce biases or distortions that compromise the validity of fluorescence measurements (Rossner and Yamada, 2004; Cromey, 2010).

### Consideration 2: avoid overexposure during image acquisition

**Figure 5.**
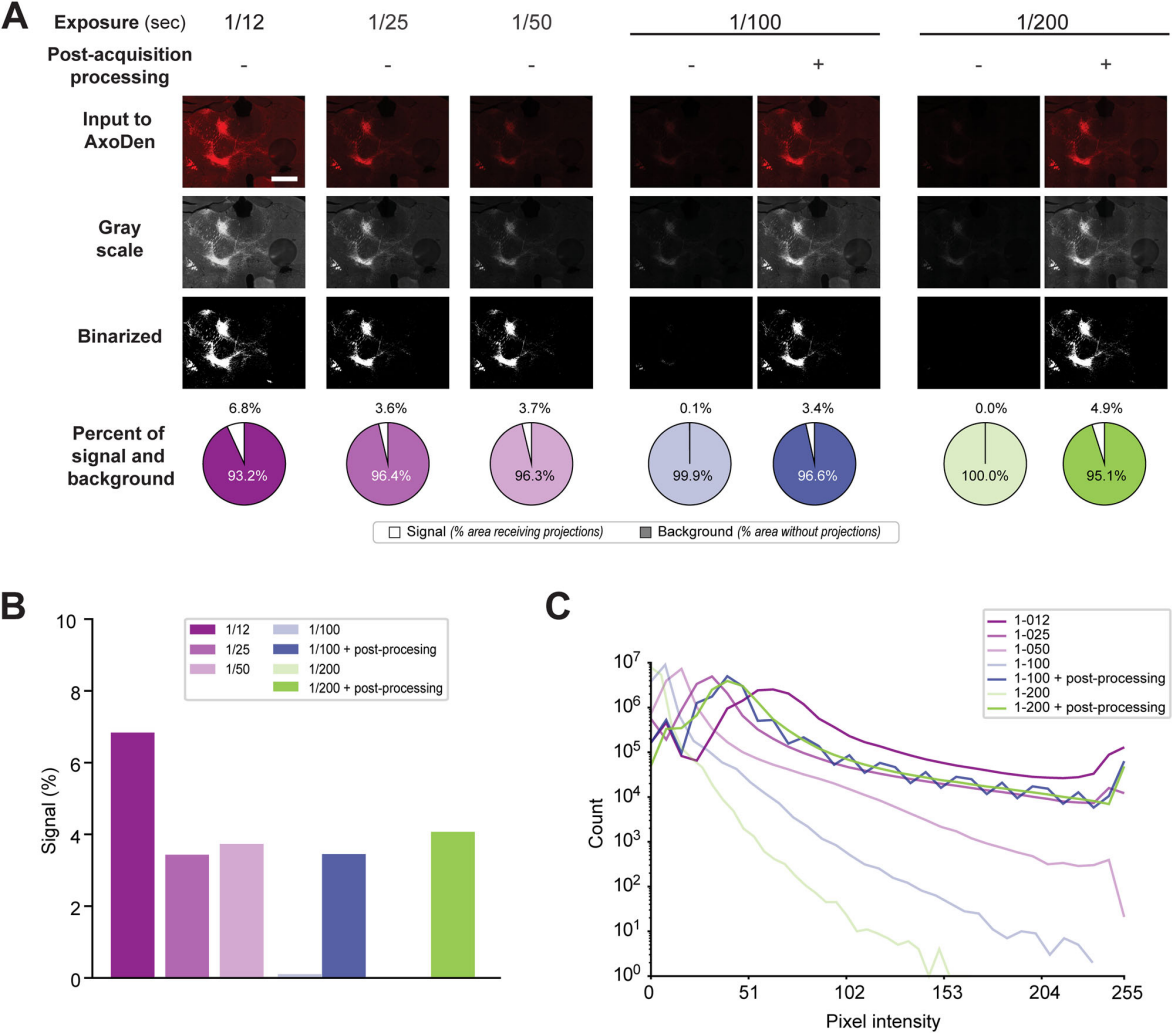
The effect of exposure time during acquisition. ***A***, Series of images of oScarlet-labeled projections from the ACC to the thalamus acquired at different exposure times, with (+) or without (−) postacquisition image processing, and their transformations and quantification of signal by the AxoDen algorithm. Scale bar, 1 mm. ***B***, Comparison of the percentage of innervation measured for each acquisition time. ***C***, Histograms of the pixel intensity values for each acquired image.

During image acquisition under a microscope, it is common practice to adjust the brightness to enhance the visibility of the fluorophore. Increasing the “Brightness”—or “Exposure”—parameter extends the duration for which the sample is illuminated by the excitation light, this is known as exposure time, thereby improving SNR. Nevertheless, excessive exposure causes two significant issues: photobleaching and image oversaturation. Photobleaching is a reduction in fluorophore emission intensity due to an irreversible photochemical reaction following extended exposure to light. Image oversaturation, or overexposure, occurs when too many pixels reach the maximum intensity value of 255, rendering those regions devoid of usable data and leading to an overestimation of signal, as we show in the example exposure time of 1/12 (Fig. 5*A*,*B*). Consequently, it is crucial to limit the number of pixels reaching their maximum value.

Thanks to the Otsu dynamic thresholding step in AxoDen, different exposure times do not affect signal detection, as seen in the example exposures times of 1/25 and 1/50 s (Fig. 5*A*,*B*). Lower exposure times, such as 1/100 and 1/200 s, do not provide enough contrast for AxoDen to segregate signal from background. However, since the pixel information remains in the image, postacquisition enhancement of brightness can achieve the same quantification values at those acquired at 1/25 and 1/50 s, indicating that information can be rescued from images with low very SNR (Fig. 5*B*). This is evident when exploring the histogram of images acquired at different exposure times (Fig. 5*C*). Decreasing the exposure times shifts the distribution of pixel intensity values toward the left, increasing the number of pixels with low values. Increasing the brightness postacquisition shifts the distribution of pixels toward the right, increasing the number of pixels with high values and allowing AxoDen to distinguish between “signal” and “background” fluorescence.

### Consideration 3 “(for cropped and masked images only)”: preserve background fluorescence in the sample area

**Figure 6.**
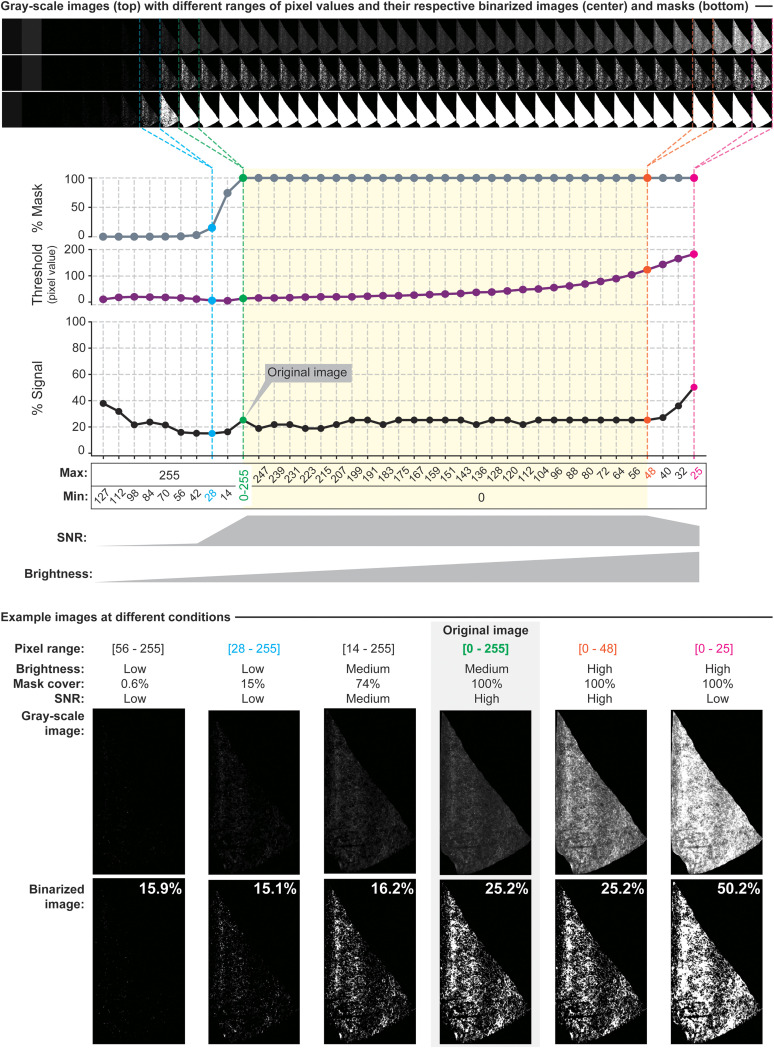
Evaluation of the robustness of AxoDen on equally spaced brightness settings. Using an example image of BLA axons projecting to ACC, the pixel range was clipped either on the low values on 10 equally spaced steps from 127–255 to 0–255 or on the high values on 20 equally spaced steps from 0–25 to 0–255. After clipping the pixel range, this was normalized to 0–255 before feeding the images to AxoDen. Clipping of low pixel values disrupted the detection of the area with tissue, generating incomplete masks that resulted in spurious quantification of signal. Once the mask covered 100% of the tissue sample, increases in brightness did not significantly alter the quantification of signal thanks to the dynamic threshold, which increased with increasing brightness. Extreme increases of brightness disrupted SNR generating unreliable results.

**Figure 7.**
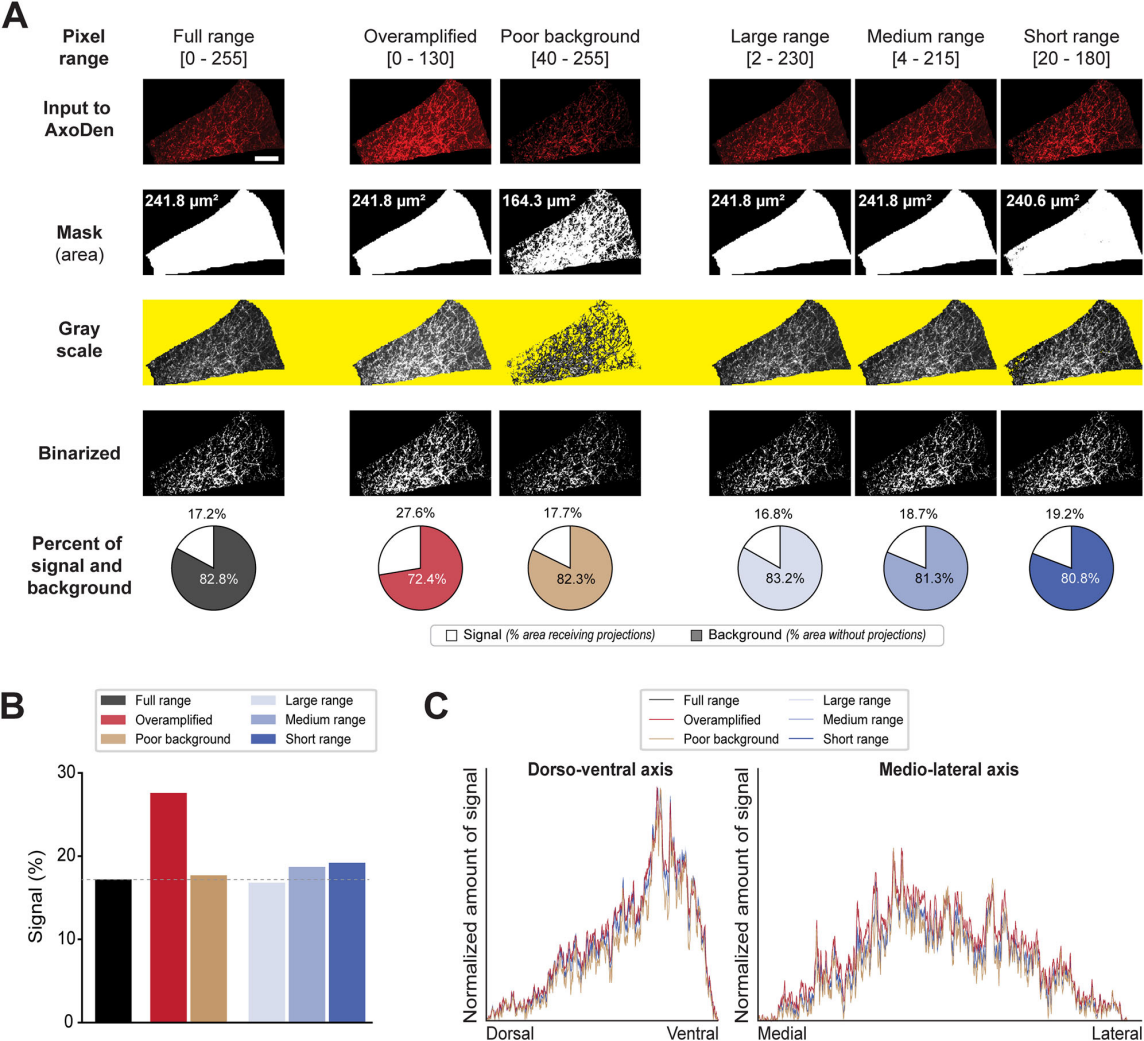
Effect of pixel ranges on the signal detection capabilities of AxoDen. ***A***, Series of images of oScarlet-labeled projections from ACC to PAG-vl with different pixel ranges and their transformations by the AxoDen algorithm. The yellow band on the grayscale row is used to visualize the transparency of the grayscale images caused by an incomplete mask. Scale bar, 100 µm. ***B***, The overall percentage of signal for each condition. The dotted line is used as a reference for the signal percentage computed for the full range image. ***C***, Normalized intensity to the maximum value of the distribution for dorsoventral and mediolateral axes.

In scenarios where the analysis algorithm processes masked images, maintaining a detectable level of background fluorescence—denoted by pixel values greater than zero—is crucial. Due to the convention of storing images in rectangular formats, areas beyond the periphery of the masked sample are assigned a pixel value of zero, indicative of an absence of data (Fig. 3*A*). Consequently, our algorithm discerns zero values as void of informational content (Fig. 3*B*). In the case where a ROI has low SNR, the user may evaluate increasing it. However, this step must be applied carefully to prevent values of zero in the area of the tissue. While AxoDen shows robustness to postacquisition adjustments in ROIs with appropriate SNR (i.e., where axons are visibly distinguishable from the background) thanks to the dynamic thresholding step, extreme modifications will provide unreliable results (Fig. 6). Figure 7 shows the effect of different postacquisition adjustments of an image where the ventrolateral region of the periaqueductal gray (PAG-vl) has been masked and cropped. Keeping the full range of pixels (Fig. 7*A*, **column 1**), AxoDen correctly classifies the “informative” pixels from the “noninformative” pixels creating a mask that covers the full area of PAG-vl (Fig. 7*A*, **column 1, second row**). Obtaining a mask that covers the full brain region (Fig. 7*A*, **column 1, third row**) confirms that AxoDen exclusively considers the pixels that fall within the mask (Fig. 3*A*) to transform the image into gray scale (Fig. 7*A*, **column 1, third row**) and compute the dynamic threshold to transform the image into a binary image (Fig. 7*A*, **column 1, fourth row**). The binary image will serve the user to confirm that there is no bleeding of the signal and to quantify the percentage of pixels that have been identified as “signal” and “background” (Fig. 7*A*, c**olumn 1, fifth row**).

Increasing SNR decreases the image’s dynamic range by clipping the ends of the pixels value spectrum. In other words, the spectrum of pixel intensity values is narrowed (Fig. 7*A*, **columns 2 to 5**) and therefore any value below the new minimum is interpreted as 0, and any value above the new maximum is interpreted as 255. However, care must be taken to prevent the overamplification of the signal by clipping the maximum values (Fig. 7*A*, **column 2**). In the example provided, the new maximum is set to 130, and therefore any pixel with a value above 130 is considered as 255. This extreme modification, which we name overamplification, leads to the artificial spread of signal intensity to adjacent pixels, falsely inflating the perceived axonal density (Fig. 7*A*, **column 2, last row**). Another crucial aspect that ensures the algorithm's accurate interpretation and analysis is the retention of minimal background fluorescence within the sample (Fig. 7*A*, **column 3**), meaning that the pixel values within the ROI remain above zero. This prevents the erroneous classification of sample as the absence of data. Increasing the minimum of the pixel range from 0 to 40 sets all pixels with values below 40 as the new 0. Therefore, all pixels that have acquired a new value of zero are considered as “noninformative” (Fig. 3*B*) and are left out of the analysis, resulting in a mask of a smaller area compared with the area of the brain ROI (Fig. 7*A*, **column 3, second row**). This in turn generates a “patchy” grayscale image of a smaller area—appreciated by the yellow background appearing throughout the PAG-vl—(Fig. 7*A*, **column 3, third row**), ultimately yielding misleading quantitative results. Nonetheless, clipping the full pixel range at different minimum and maximum levels yielding images with new pixel ranges of different lengths (Fig. 7*A*, **columns 4 to last**) shows that the algorithm is robust to variations of pixel ranges (Fig. 7*A*, **columns 4 to last**). However, when the clipping is so severe that the background fluorescence reaches values of zero and the mask does not cover the entirety of the brain region, an overestimation of the signal occurs (Fig. 7*A*, **last column**). Comparison of the signal percentage in the previous conditions shows that images with overamplified signal yield the highest overestimation of projections (Fig. 7*B*). However, variation of the signal percent is kept low between the different pixel ranges and the normalized intensity across both the dorsoventral and the mediolateral axes do not show large differences between conditions (Fig. 7*C*). The robustness of AxoDen to acquisition settings (Fig. 5) as well as to postacquisition adjustments (Fig. 7), which is provided by the Otsu dynamic thresholding method, prevents inter-researcher variation (Fig. 1), making AxoDen a tool easily accessible to researchers at all career levels.

### Consideration 4 (*Optional*): rotate the image for accurate spatial distribution of axes

**Figure 8.**
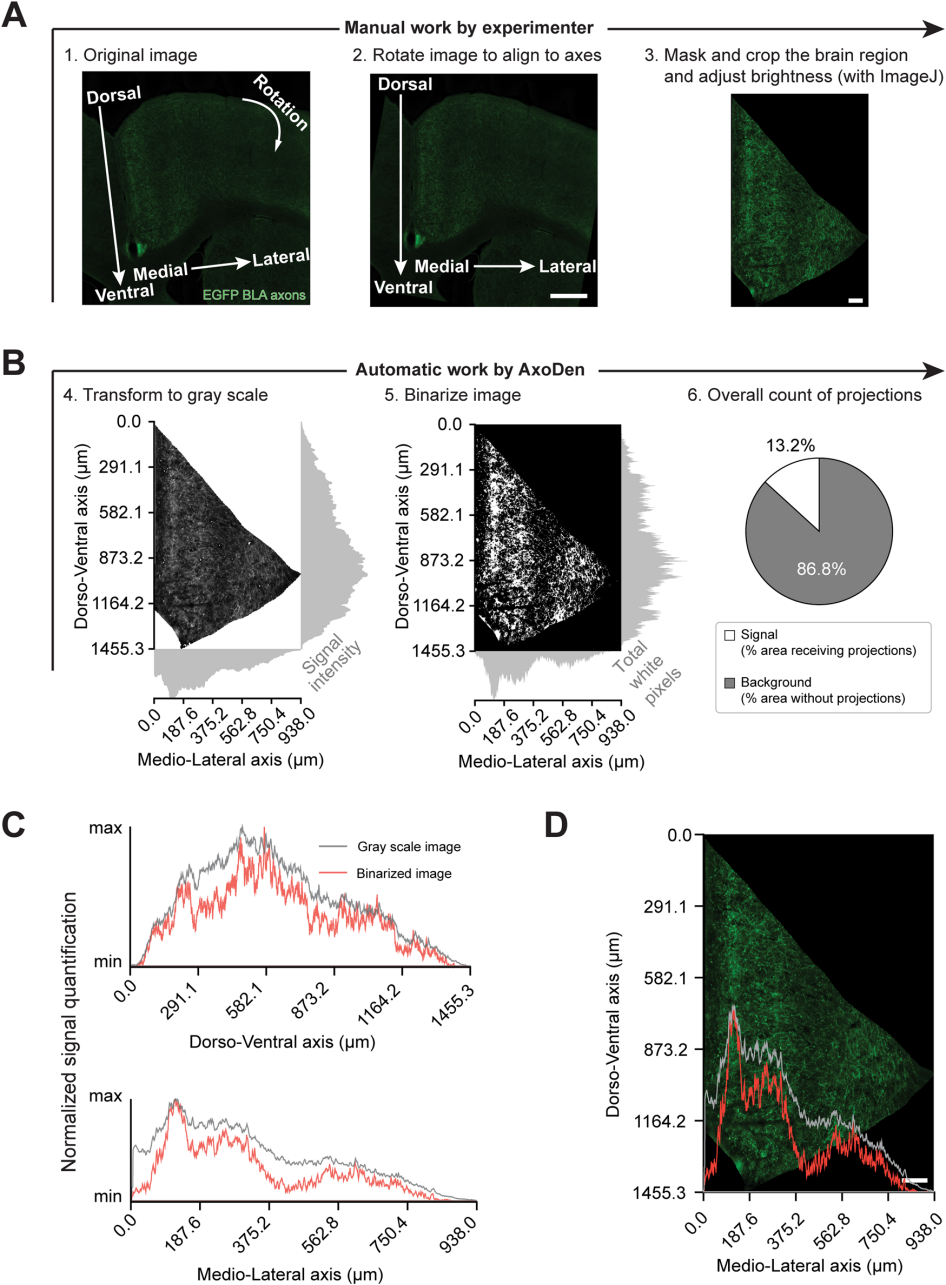
Spatial distribution of projections along the *x* and *y* axes in the ACC. ***A***, Manual steps users need to follow to first rotate (A1-2) an image to align the dorsoventral and the mediolateral axes to the *y* and *x* axis and then mask and crop the region of interest (A3). Scale bar (Step 2), 500 µm. Scale bar (Step 3), 100 µm. ***B***, Automatic AxoDen steps where the signal intensity is measured along the axes in grayscale images (B4), and the total of white pixels in the binarized images (B5). Lastly, the percentage of white pixels, referred to as “% area receiving projections” is computed independently of the axis (B6). ***C***, Amount of signal normalized to the maximum value of the distribution along the dorsoventral and the mediolateral axes comparing the values obtained from grayscale and binarized images. ***D***, Overlay of the signal intensity on the mediolateral axis with the cropped image to visually show that the quantification along the axis of binary images better follows the axonal distribution compared with the quantification of signal intensity from grayscale images. Scale bar, 100 µm.

In contexts requiring detailed spatial characterization, such as the evaluation of innervation profiles across cortical layers, the user can opt to rotate the image, before masking and cropping the region of interest, and feed the resulting image to AxoDen (Fig. 8). This alignment ensures that the fluorescence quantification accurately reflects the spatial distribution of interest, thereby yielding data that are both reliable and biologically meaningful. With the example of projections from BLA to ACC (Wojick et al., 2024), the user rotates the image to align the dorsoventral and mediodorsal axes with the y and x axes, respectively. Then, proceeds to mask and crop the ACC (Fig. 8*A*) to later feed the resulting image into AxoDen. The AxoDen algorithm first converts each input image to gray scale and then creates a corresponding binary image. At each stage, AxoDen quantifies the axonal signal along both the *x* and *y* axes, as described in section “Interpretation of the figures created by AxoDen.” Briefly, in the grayscale image, the axonal signal is calculated as the mean pixel intensity within a predefined range, whereas in the binary image, only the white pixels, which are considered as “signal,” are counted (Fig. 8*B*). Because the grayscale calculation includes background fluorescence, the SNR is typically lower than in the binary image, where background pixels are excluded (Fig. 8*C*). Overlay of the intensity profiles of the mediolateral axis with the cropped ACC shows that the profile obtained from the binarized image better recapitulates the innervation pattern of the different cortical layers of ACC (Fig. 8*D*). Therefore, only the information of the binarized image should be taken into consideration for this analysis, given that in the background fluorescence of grayscale images occludes the innervation patterns of different cortical layers.

### Consideration 5: adhere to the algorithm’s naming convention

Any algorithm operates through a sequence of systematically arranged instructions. Thus, adhering to the AxoDen’s naming convention is crucial to guarantee that data from each image are accurately extracted and categorized for effective analysis. AxoDen relies on the file name to derive essential details about each image, using the underscore symbol (“_”) as a delimiter to segregate distinct information details within the name. Subsequently, it categorizes the segmented information based on the position it occupies. The first position is designated as the subject identifier, the second position denotes the brain region, and the third position represents the experimental group. In cases where there is only one experimental group, write the same word (i.e., “group”) in the third position of the file name for all the images of the given experiment. For instance, in a filename like:

mouse123_AnteriorCingulateCortex_control_additional-information.tif,

mouse123 is interpreted as the subject identifier, AnteriorCingulateCortex specifies the brain region, and control identifies the experimental group. The algorithm disregards any text succeeding the experimental group identifier, providing a series of positions for supplementary details about the image that does not influence the core data extraction process. Furthermore, the algorithm recognizes and processes images specifically in the .tif format. Consequently, ensuring that all images prepared for analysis are saved in this format is imperative.

### Defining an adequate SNR

An adequate SNR is crucial for reliable axonal segmentation, as AxoDen relies on dynamic thresholding using the Otsu methods to distinguish labeled axons from background in a wide variety of imaging conditions. Conceptually, adequate SNR means that the pixel intensity distribution of labeled axons is sufficiently separated from baseline noise or background fluorescence, enabling a clear cutoff between “signal” and “nonsignal.” While SNR can be quantified in various ways (e.g., ratio of mean signal intensity to background standard deviation), experiments differ widely in fluorescence markers, exposure settings, and tissue properties. As a result, AxoDen does not enforce a specific numeric threshold for “adequate” SNR. Thus, we recommend users inspect the control plots and assess whether the detected axons in the binarized image are discernible by eye in the grayscale images.

### Use of AxoDen in nonfluorescent staining

**Figure 9.**
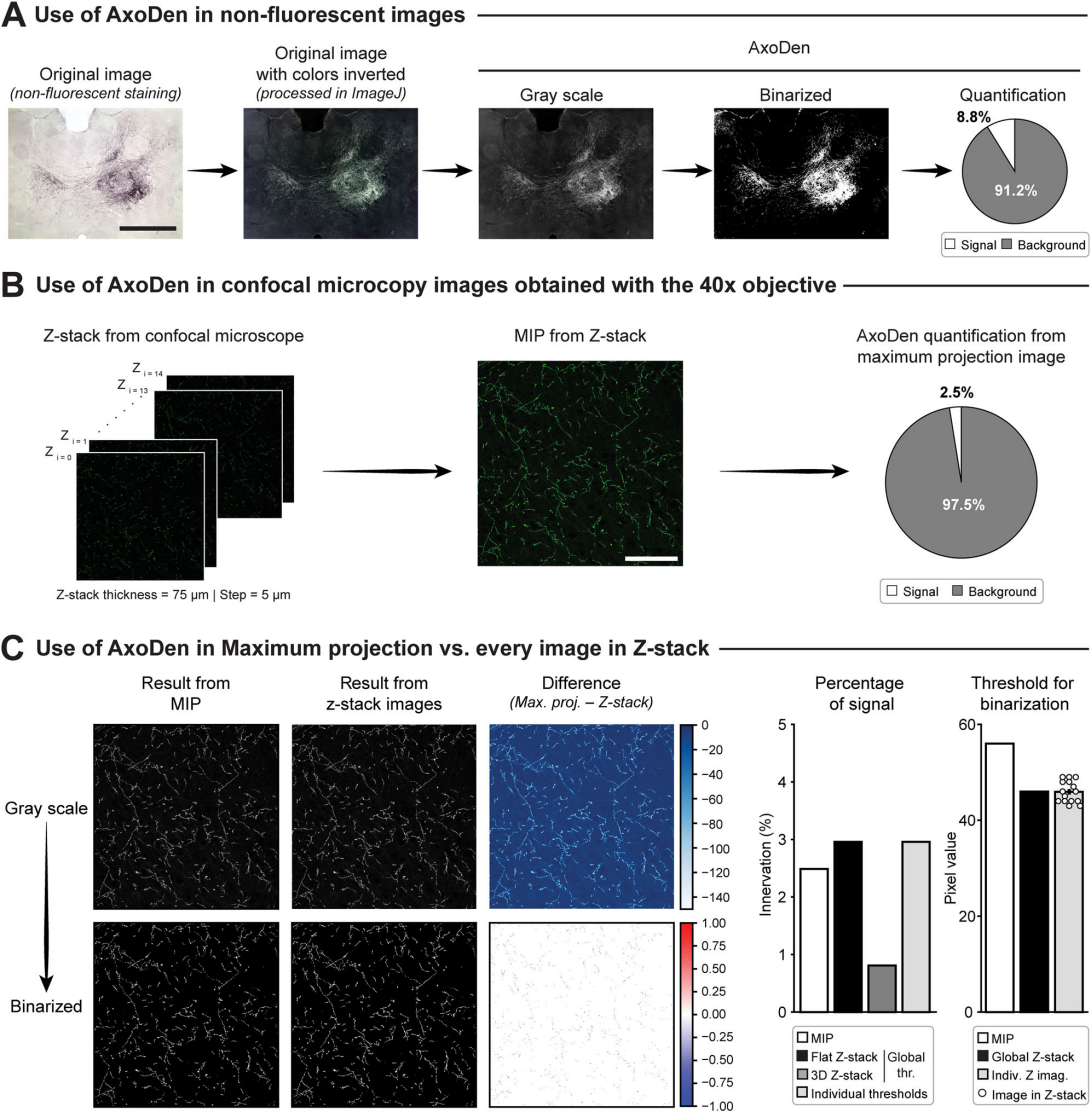
Use of AxoDen in nonfluorescent images and confocal micrographs. ***A***, Example of the processing and AxoDen quantification of a nonfluorescent image. Scale bar, 1 mm. ***B***, Example of the use of AxoDen in a confocal image acquired with the 40× objective. Scale bar, 100 µm. ***C***, Comparison of the results provided by AxoDen when processing the MIP image or each single image of the *Z*-stack. Left, Images in gray scale and binarized resulting from the maximum projection obtained by the microscope software, the *Z*-stack images, and the difference between MIP and the *Z*-stack in 2D. Right, Bar plots showing the percentage of innervation of the area and the threshold obtained to apply the binarization step.

Optical microscopy tracing has often relied on nonfluorescent tracers such as the biotin derivative, biocytin, which is detected via streptavidin and colorimetric development [e.g., 3.3′-diaminobenzidine tetrahydrochloride (DAB)]. This process results in darkly stained axons that stand out against the lighter background, allowing clear pixel classification and the segmentation of labeled axons. To test AxoDen on nonfluorescent stainings, we used an image of a coronal brain section containing the intralaminar thalamic nuclei from Fillinger et al. (2018), in which mice were injected with *Phaseolus vulgaris* leucoagglutinin in ACC and revealed with DAB to study the efferents of this brain region (Fig. 9*A*). First, colors were inverted in ImageJ (Edit > Invert), and then, the result was saved as a TIFF file and fed to AxoDen. AxoDen classified signal from nonsignal regions, quantifying the percentage of the image containing axons. This confirms that AxoDen can be used on nonfluorescent stainings.

### Use of AxoDen in confocal images obtained at higher magnifications

We tested AxoDen in 16 bit images acquired of the prefrontal cortex with a classical confocal microscope using the 40× objective (Fig. 9*B*), yielding a 75-µm-thick *Z*-stack with 5 µm steps. The norepinephrine transporter of axons of the locus ceruleus was labeled with a green fluorescent protein. First, we generated the maximum intensity projection (MIP) of the *Z*-stack and processed it with AxoDen (Fig. 9*B*). We then compared the results to those obtained by analyzing each slice of the *z*-stack individually (Fig. 8*C*). For 3D analysis, each 2D slice was converted to gray scale and then binarized using a global threshold derived from the entire stack. We also created a flattened version of the gray scale and binarized *Z*-stacks (the “flat *Z*-stack”) by selecting the maximum pixel intensity along the *z* axis for each XY-coordinate. To compare the MIP with the flat *Z*-stack, we generated difference images by subtracting the flat *Z*-stack from the MIP. In a grayscale form, the difference was near zero in background regions; negative values indicated brighter signal in the flat *Z*-stack than in the MIP. For binarized images, the differences were minimal, with a few scattered pixels at −1, indicating slightly more signal in the flat *Z*-stack. Next, we quantified the percentage of labeled axons in the 3D *Z*-stack, the flat *Z*-stack, and the MIP (Fig. 8*C*, bar plot “percentage of signal”). The MIP and flat *Z*-stack showed similarly high percentages of innervation, whereas the 3D *Z*-stack had a lower percentage due to inclusion of more background (black pixels). This underscores the importance of using a consistent image format for AxoDen analyses. Nevertheless, the MIP produced results comparable to the full *Z*-stack, and using individual thresholds for each slice did not significantly alter the final outcome relative to a single global threshold (Fig. 8*C*, bar plot “threshold for binarizatoin”).

In summary, these findings indicate that (1) AxoDen can be effectively applied to 16 bit images acquired with a 40× objective on a confocal microscope and (2) analyzing 2D maximum projections can provide quantification results comparable to those obtained from full 3D *z*-stacks while offering reduced computational demands.

## Discussion

Axonal quantification has been a challenging metric. Until recently, semiquantitative approaches have dominated large-scale neuroanatomical studies. For example, Fillinger et al. (2018) categorized axonal innervation levels into qualitative bins ranging from “no labeling” to “heavy labeling.” While such strategies remain valuable for broad overviews, today’s data-rich environment favors more fully quantitative methods. Consequently, many researchers resorted to intensity-based measurements for axons. Yet, fluorescent intensity measures carry substantial variability and do not always reflect true axonal density or coverage. Automated segmentation and thresholding methods have revolutionized related fields—such as cell counting, tractometry in MRI, and retinal layer segmentation—but axonal analysis has lagged behind because of the complex, branching morphology inherent to axons. Hence, an intuitive, automated tool that applies threshold-based segmentation to images without expensive instrumentation or advanced programming skills has been a long-standing need.

Here we introduce AxoDen (Fig. 1*A*), a platform to streamline, standardize, and speed up the quantification of axonal innervation of defined brain ROIs. AxoDen is robust against different exposure times (Fig. 5) and diverse postacquisition adjustments thanks to the dynamic thresholding step (Figs. 6–7). This robustness decreases the variability in the measurements taken by different researchers (Fig. 1 *C*–*G*). AxoDen additionally provides a more accurate representation of brain area innervation as it quantifies the distribution of axonal projections in the *x* and the *y* axes (Fig. 8). Furthermore, given that this method relies on the range of pixel values of one single channel to segment the image, the color of the fluorophore does not impact the quality of the quantification (Fig. 3). In principle, threshold-based segmentation rests on the premise that signal and background intensities are sufficiently distinguishable—a concept well established in morphological image analysis (Cardullo and Hinchcliffe, 2013; Uchida, 2013). By converting raw fluorescence values into a binary representation of “signal” versus “nonsignal,” AxoDen captures the morphological presence of axons while mitigating confounds arising from raw intensity fluctuations or uneven illumination. Thus, our method promises to make high-quality analysis of axonal innervation accessible to every laboratory and researcher irrespective of training experience and to enhance the precision of axonal density quantification for any image of fluorescently labeled axonal projections taken with an objective of 20× or above for any animal species. It is important to consider, however, that such robustness can only be achieved if there is consistency in the type of images fed to AxoDen.

### How AxoDen contributes to the improvement of axonal quantification

The challenge of axonal innervation analysis lies on the large differences in axonal density of adjacent brain regions that are unavoidably imaged together (Fig. 4 and compare CL with MD—see anatomy of CL and MD in Fig. 1*A*). If interested in the information of both brain regions, the use of AxoDen allows the researcher to image CL and MD at once, saving time and preserving the fluorophore quality. In such case, to prevent overexposure of CL projections, acquisition intensities must remain low, which results in MD being underexposed. Because AxoDen does not quantify fluorescence intensity and only segments the image into “signal” and “nonsignal,” after masking CL and MD separately (example of CM masking in Fig. 1*A*), the researcher can carefully increase the SNR without causing large alterations in the final quantification (Figs. 4–7). This postacquisition adjustment increases the dynamic range of the masked and cropped MD allowing AxoDen to find a threshold for segmentation, subsequently binarize the image, and proceed with quantification. Furthermore, because the masked and cropped brain ROIs are saved as TIFF files and AxoDen provides control figures for each ROI where each step of the algorithm is plotted (example in Fig. 2D), the researcher, and anyone interested, can always confirm that no overexposure or extreme modification of the image has been performed.

### AxoDen potential to standardize and streamline axonal quantification

There are many algorithms developed in the past years that have opened their source code in repositories such as GitHub. However, a common challenge in biology research is that, very often, researchers have not learned how to use these repositories nor how to write scripts for analysis. This is why we believe that making the code accessible is not enough. For this reason, we have created a stand-alone GUI and a web application that anyone, independently of their coding skills, can use. Furthermore, we have purposefully kept AxoDen simple to understand because only by being able to understand the steps of the analysis, the interpretation of the results can be accurate and closer to the actual biological meaning. As a result, by replacing the use of different parameters and workflows with a single set of robust and evaluated parameters, and an easy-to-understand workflow, AxoDen has the potential to increase reproducibility between and within laboratories.

### Expertise required to implement AxoDen

AxoDen is versatile, capable of analyzing any brain region independently of its shape, fluorophore used, or animal species. It is specifically designed to accommodate users with varying levels of coding expertise, thereby broadening its applicability across diverse research contexts. As such, users are not required to have advanced data analysis skills to utilize the software effectively. However, a fundamental understanding of neuroanatomy for the specific species under investigation is essential. This knowledge is crucial to (1) accurately identify and select the targeted brain region for analysis and (2) correctly orient the image to align with the relevant axes, ensuring that the derived *x* and *y* axis signal quantification profiles are biologically significant. Additionally, users should be familiar with the basic image analysis software, such as the widely used ImageJ, to adjust the SNR. Enhancing this ratio has the potential to improve the clarity and distinguishability of the signal, facilitating more accurate and reliable quantification of axonal density, for those images with low exposure times.

### Limitations

AxoDen provides notable improvements in the quantification of axonal projections, yet it has limitations that need attention. First, while AxoDen effectively quantifies axonal projections, it cannot distinguish axons from cell bodies. As a result, neuronal somas might be erroneously included in the axonal counts, potentially biasing the outcome. As with somas, AxoDen is unable to identify and exclude artifacts like bubbles or dust. To address these challenges, a recommended preprocessing step involves manual identification and removal of neuronal cell bodies and artifacts from the images before proceeding with the analysis. Although this step introduces additional time investment, it substantially enhances the precision of the axonal quantification. If the user has one spare channel that can use as a reference, the availability of AxoDen source code makes it suitable to integrate with artifact removal algorithms like the one described in DEFiNE (Powell et al., 2019). Another limitation is the need of enough high resolution to identify axons. AxoDen has been developed using images obtained with a 20× objective because this objective lens, which strikes a balance between field of view and resolution sufficient for identifying axonal shapes and assessing their density and distribution. Given that AxoDen is based on the segmentation of axons, only those lenses with magnifications where axons can be segmented are recommended (Fig. 9*B*). Utilizing images obtained with a 4× objective lens is not advised due to the lower resolution, which impedes the accurate differentiation of axons from other structures, thereby compromising the integrity of the analysis. For studies necessitating the use of 4× magnification—often aimed at broader regional assessments—we suggest adhering to traditional mean fluorescence intensity measurements, despite their known limitations. This recommendation is made with the understanding that the insights gained from such analyses at lower magnifications serve different research objectives, primarily related to general patterns of innervation rather than detailed quantifications of axonal density and distribution. Lastly, it is noteworthy that edge cases, such as ROIs with high brightness but no axons, can yield erroneous results due to the Otsu dynamic thresholding used in AxoDen. If these ROIs are adjacent to highly innervated brain regions, the brightness of the edge case ROI can be reduced to reflect the true nature of the ROI. Even though AxoDen is robust to acquisition and postacquisition variations of images with adequate SNR (where labeled axons clearly stand out from the background), any extreme modification will inevitably output wrong results. We encourage all users of AxoDen to carefully evaluate the control plot of each ROI to ensure that AxoDen correctly segmented the pixels, independent of the ROI’s SNR.

## Synthesis

Reviewing Editor: Michael Michaelides, NIDA-NIH Decisions are customarily a result of the Reviewing Editor and the peer reviewers coming together and discussing their recommendations until a consensus is reached. When revisions are invited, a fact-based synthesis statement explaining their decision and outlining what is needed to prepare a revision will be listed below. The following reviewer(s) agreed to reveal their identity: Hiroyuki Ichijo.

Reviewer 1

In this study, the authors developed AxoDen, a workflow to quantify the area of axonal signals in fluorescence images. In the first step, z-stacked 2D images are cropped based on the reference atlas. The images are then binarized using the Otsu method. Finally, the density of signal-positive areas is calculated. Figures 4 and 5 show the results for example images acquired under variable conditions.

Currently, neuroscientists are trying to obtain comprehensive data in an unbiased manner. State of the art analysis pipelines are mostly designed for whole brain analysis. AxoDen is designed for conventional analysis of 2D wide-field fluorescence images. In my opinion, most of the procedures in AxoDen are already possible with ImageJ/Fiji, and some of the steps are not so elegant. Most people use ImageJ/Fiji macros to perform the same analyses. In particular, the Otsu method is critical to the consistent performance of AxoDen; however, the Otsu method is already implemented in the standard ImageJ functions (Image > Adjust > Threshold). It would be nice to streamline everything with a simple GUI, but the technical advance in this paper is thin.

In addition, some of the steps recommended in this paper are inappropriate or even violate the ethics of quantitative image analysis, as described below. I don't understand why the results between two experimenters are so different. Manual steps in image analysis should be minimized (except for the cropping part, which would be difficult to automate).

Major

1) The authors recommend to adjust the brightness/contrast of the images before quantitative analysis. This function is useful to make the images look better for presentation purposes, but it is totally inappropriate for quantitative analysis. Manual and subjective manipulation of quantitative data is not allowed.

2) Since the axons are distributed in 3D, the density should be quantified in 3D or in a single 2D slice. The authors recommended z-stacking images for multiple slices; however, AxoDen values after this procedure would no longer be proportional to the actual density of axons. For example, if axons are located on one side of the volume, x-y and x-z stacks will produce different values in the AxoDen pipeline.

3) It is inappropriate to use different thresholds for different images of the same series taken under the same condition. The Otsu method should work well for ROIs with many axons, but probably not for ROIs without axon signals. Once the threshold is determined (for the entire image set or for representative images with many axons), the same threshold must be applied to the entire image set for binarization.

Minor

4) Line 155. I don't think this procedure is new. Only the GUI is different.

5) Line 172. I don't think it is common to measure ROIs with a rectangular shape. With ImageJ/Fiji, we can easily draw ROIs in any shape and quantify both area and mean intensity. It is unfair to use results from different shapes of ROIs to compare AxoDen vs. Mean Intensity.

6) Line 177. The authors can't draw conclusions with n=2 comparisons. This paper also lacks statistical analyses.

7) Line 208. This is useless and inappropriate. We can properly define the threshold without this step.

8) Line 290. It is more common to take images at 12-bit or 16-bit for quantitative image analysis.

9) Line 314. This is not so smart. We can use the ImageJ ROI manager to avoid this problem.

10) Scale bars are missing in most images.

11) There is no information about the AAV vectors used in the study.

12) The graphs in Figure 5 and 6 are missing units. It is unclear how the intensity was normalized.

Reviewer 2

The manuscript is a concise and clear description of a new and useful method. Although we cannot actually test the performance of AxoDen here, so the true evaluation will be left to the User, this reviewer understood the relevance of the manuscript to be delivered to the User.

On the other hand, the manuscript emphasized the advantages from the User's point of view, so there was little description of the technical advantages as the creator, and I would have liked to know a litle more about this in some detail. I would have liked to have received a technical explanation of the part where AxoDen contributed to the improvement compared to the conventional method, even if it was only a short sentence.

Could you also describe the amount of future possibilities to detect and technically exclude limitation No.1, and briefly describe the technical reasons for limitation No.2 (optimisation for ×20 lens)? What about the possibility of creating a generalised method that can be used for other objective lenses?

In addition, the z-stack of the image acquisition (Keyence BZ-X810, in the mansusript the name was wrong, line 141) in this experiment seems to be a software approximation and not equivalent to the confocal optical section in exact sense, is that correct? If so, is AxoDen compatible for use with confocal optical sections? Furthermore, is it compatible with images acquired by traditional optical microscopy tracing methods such as Biocytin with DAB staining?

Minor points

(1) Cities and nations for many companies are not described.

(2) Several anatomical abbreviations, although common, are used without definition (lines 96, 97, BLA, CM) or before definition (ACC, line 76), which needs corrections.

(3) The name of the university or laboratory to which the author supposedly belongs was given. lines 64, 144, and

github, etc. were also listed, and all are shown.

(4) Regarding the comparison of time and variability, how about stating "n" in the text? lines 175, 178.

## Author Response

We sincerely thank the reviewers for their feedback on our manuscript titled "AxoDen: An Algorithm for the Automated Quantification of Axonal Density in defined Brain Regions ", which have greatly helped us to refine and improve our work. In response, we have carefully addressed each point and made significant revisions to strengthen the manuscript. Below, we provide a detailed account of the changes made in response to the reviewers' comments.

Below, we have reiterated the original reviewer comments in blue text, and our responses are immediately preceding in black text with new figures and/or new text provided in this response letter. Within the main text document, all new text is highlighted.

Overall comment: Synthesis of Reviews for manuscript Computational Neuroscience Model Code Accessibility Comments for Author (Required):

Please include a statement in the Materials and Methods section, under the heading "Code Accessibility", indicating whether and how the code can be accessed, including any accession numbers or restrictions, as well as the type of computer and operating system used to run the code.

We have added the section "Code Accessibility" in the Methods section.

Reviewer 1 In this study, the authors developed AxoDen, a workflow to quantify the area of axonal signals in fluorescence images. In the first step, z-stacked 2D images are cropped based on the reference atlas. The images are then binarized using the Otsu method. Finally, the density of signal-positive areas is calculated. Figures 4 and 5 show the results for example images acquired under variable conditions.

Currently, neuroscientists are trying to obtain comprehensive data in an unbiased manner. State of the art analysis pipelines are mostly designed for whole brain analysis. AxoDen is designed for conventional analysis of 2D wide-field fluorescence images. In my opinion, most of the procedures in AxoDen are already possible with ImageJ/Fiji, and some of the steps are not so elegant. Most people use ImageJ/Fiji macros to perform the same analyses. In particular, the Otsu method is critical to the consistent performance of AxoDen; however, the Otsu method is already implemented in the standard ImageJ functions (Image > Adjust > Threshold). It would be nice to streamline everything with a simple GUI, but the technical advance in this paper is thin.

Indeed, these are analysis steps that can be performed in ImageJ via several different macros that must be independently known about and then downloaded/installed and then data outputs moved between them. We are unaware of a published manuscript detailing a protocol to "streamline" such methods. Thus, we are not claiming to advance the state-of-the-art in the analysis, which would require much more complicated methods. We are trying to lower the barrier to axonal analysis for researchers to take advantage of these open-source methods/protocols, which provide a standardized the analysis of axonal innervation for fast and reliable every-day analysis at all levels of technical capabilities and expertise. Therefore, we argue that the potential impact of AxoDen to improve the workflow of some neuroscience labs outweighs technical "thinness" of the underlying analysis steps.

The open access and high accessibility of AxoDen places our platform in a unique position to standardize and streamline the analysis of axonal innervation. By replacing the use of different parameters and workflows with a single set of robust and evaluated parameters, and an easy-to-understand workflow, AxoDen has the potential to increase reproducibility between and within laboratories. We have expanded this clarification in the Discussion section under "AxoDen potential to standardize and streamline axonal quantification" (line 533).

AxoDen potential to standardize and streamline axonal quantification.

There are many algorithms developed in the past years that have opened their source code in repositories such as GitHub. However, a common challenge in biology research is that, very often, researchers have not learned how to use these repositories nor how to write scripts for analysis. This is why we believe that making the code accessible is not enough. For this reason, we have created a stand-alone GUI and a web application that anyone, independently of their coding skills, can use. Furthermore, we have purposefully kept AxoDen simple to understand because only by being able to understand the steps of the analysis, the interpretation of the results can be accurate and closer to the actual biological meaning. As a result, by replacing the use of different parameters and workflows with a single set of robust and evaluated parameters, and an easy-to-understand workflow, AxoDen has the potential to increase reproducibility between and within laboratories.

In addition, some of the steps recommended in this paper are inappropriate or even violate the ethics of quantitative image analysis, as described below. I don't understand why the results between two experimenters are so different. Manual steps in image analysis should be minimized (except for the cropping part, which would be difficult to automate).

We agree that there will always be inter-experimenter variability in any scientific method of data collection and/or analysis. We acknowledge this inevitable variability and provide AxoDen as an improved standardized tool with comprehensive documentation to stream the process, and improve the reliability and reproducibility of axonal quantification, thereby advancing the overall integrity of scientific research. The reviewer has correctly identified the primary source of inter-experimenter variance in AxoDen - namely, the manual image cropping step that can result in small differences in free-hand drawing of the ROI (i.e., the exact pixels framing the putative boarder of a brain region can be different). As the reviewer notes, this specific step in the AxoDen protocol is very difficult to automate at this time. We hope that future iterations can build from the AxoDen platform to reduce these small discrepancies.

Nonetheless, we now provide new direct comparisons between AxoDen vs. traditional square ROI-based mean intensity measurements in updated Figure 1C, 1D and 1E showing that AxoDen significantly reduces the inter-experimenter variance across analyzed animal datasets (Fig. 1C, P = 0.05) and within specific brain regions (Fig. 1D,E; Mann-Whitney U-Test, P = 0.0002):

Figure 1 │ AxoDen overview and comparison to Mean Intensity method. C) Overall variance between researchers for AxoDen and Mean Intensity. D) Variance between experimenters when using AxoDen and Mean Intensity for multiple brain regions. E) Distribution of the variance between researchers for each method. Mann-Whitney U test revealed AxoDen has an overall lower variance than Mean intensity. **, p-value < 0.001.

Major 1) The authors recommend to adjust the brightness/contrast of the images before quantitative analysis. This function is useful to make the images look better for presentation purposes, but it is totally inappropriate for quantitative analysis. Manual and subjective manipulation of quantitative data is not allowed.

We believe there is a fundamental misunderstanding with the AxoDen method, namely we are not quantifying fluorescence intensity. We are segmenting the pixels of the image into a binary: signal (axons are present/exist in the image) or no signal (axons are not present/exist in the image). Therefore, we strongly disagree with the ethical suggestions of this comment.

Indeed, there are no acquisition parameters that fits all because of huge variability in virus expression, antibody penetration and binding, quality of PC screen for image acquisition. Therefore, these parameters are typically chosen arbitrarily (e.g. 2 SD above background, etc.), and quantification of fluorescence intensity can indeed be subject to problematic post-acquisition manipulation. However, we are not quantifying fluorescence intensity; we are segmenting the pixels of the image into signal or no signal. We absolutely agree with Rev. 1 that altering images to yield quantitatively different results for image intensity/density should not be done under any circumstance, and we are in no way advocating for such behavior in this manuscript.

Given that acquisition parameters can be so subjective and variable across labs and different experiments, we directly compared the results of applying AxoDen to the same brain region acquired at different exposure times (new Figure 4). AxoDen was robust to different exposure times. Long exposure times during acquisition overestimated the percentage of signal. However, long exposure times cause fluorophore bleaching and are not recommended, which is stated in the manuscript text.

The challenge of axonal innervation analysis is the large differences of fluorophore expression between adjacent brain regions that are unavoidably imaged together (see Figure 4 and compare the thalamic subnuclei centrolateral (CL) with the mediodorsal (MD) - see anatomy of CL and MD in Figure 1A). To prevent overexposure of CL projections, acquisition intensities should remain low, meaning that MD is underexposed. The resulting low dynamic range caused by underexposure can be recovered by increasing the brightness post-acquisition (Figure 5). Because we are not quantifying fluorescence intensity, increasing the brightness will only lead to an increase in the dynamic range, allowing AxoDen to find a threshold for segmentation. This has been added as a Consideration (Consideration 1, new figure 4) and included in the discussion under the subsection "How AxoDen contributes to the improvement of axonal quantification".

Consideration 1: Large brightness differences in adjacent brain regions may require post-acquisition adjustments.

Adjacent brain regions may exhibit significantly different levels of innervation (Fig. 4A). Consequently, during image acquisition, exposure settings are typically optimized for regions with higher fluorescence to prevent irreversible fluorophore bleaching. This approach ensures that areas with sufficient projections have an adequate dynamic range for AxoDen to effectively distinguish signal from background noise (Fig. 4B, CL and MD). However, regions with fewer projections present a reduced dynamic range and poor signal-to-noise ratio (Fig. 4B, VM raw image). Without post-acquisition adjustments after cropping the images, the signal intensity of axons projecting to the VM region remains too low for AxoDen to detect (Fig. 4B, VM raw image). By adjusting the pixel intensity range post-acquisition using image processing software like ImageJ, the signal-to-noise ratio can be enhanced. This enhancement enables AxoDen to accurately segment "signal" from "non-signal" pixels, successfully detecting the axons in the VM region (Fig. 4B, VM SNR enhanced).

How AxoDen contributes to the improvement of axonal quantification.

The challenge of axonal innervation analysis lies on the large differences in axonal density of adjacent brain regions that are unavoidably imaged together (see Figure 4 and compare CL with MD - see anatomy of CL and MD in Figure 1A). If interested in the information both brain regions, the use of AxoDen allows the researcher to image CL and MD at once, saving time and preserving the fluorophore quality. In such case, to prevent overexposure of CL projections, acquisition intensities must remain low, which results in MD being underexposed. Because AxoDen does not quantify fluorescence intensity and only segments the image into "signal" and "non signal", after masking CL and MD separately (example of CM masking in Figure 1A), the researcher can modify the pixel range for the underexposed brain region to increase the brightness without causing large alterations in the final quantification (Figure 5). This post-acquisition adjustment increases the dynamic range of the masked and cropped MD allowing AxoDen to find a threshold for segmentation, subsequently binarize the image and proceed with quantification. Furthermore, because the masked and cropped brain ROIs are saved as TIFF files and AxoDen provides control figures for each ROI where each step of the algorithm is plotted (example in Figure 2D), the researcher, and anyone interested, can always confirm that no overexposure or extreme modification of the image has been performed.

2) Since the axons are distributed in 3D, the density should be quantified in 3D or in a single 2D slice. The authors recommended z-stacking images for multiple slices; however, AxoDen values after this procedure would no longer be proportional to the actual density of axons. For example, if axons are located on one side of the volume, x-y and x-z stacks will produce different values in the AxoDen pipeline.

A single 2D slice (or "step", which is user-defined volume) obtained from a z-stack (also a user-defined variable) will only contain bits and pieces of axons, which may have less biological information than the entire stack. Hence, why we, in this one example, elected to perform quantification of z-stacked images and made the recommendation. Of course, users of AxoDen can choose to z-stack or not according to their experimental designs. Obviously, performing either x-z or x-y stacking will generate different results and AxoDen takes advantage of this property. By aligning the x and the y axis of an image with the medio-lateral and the dorso-ventral axes of the brain, respectively, AxoDen can provide information on the distribution of axons on both axes and therefore on the innervation of different cortical layers in ACC (updated Figure 7). The spatial resolution will depend on the parameters of image acquisition, and if the user wishes to compute a continuous innervation profile in the anterior-posterior axis, the user should collect sagittal instead of coronal brain slices.

To further address this concern, we have added the section "Use of AxoDen in confocal images obtained at higher magnifications" and updated the new Fig 8. This section shows that AxoDen is performing robustly at higher magnifications and in images obtained at a confocal microscope. Furthermore, we show that applying the AxoDen image processing steps on each single image of the z-stack generates the same results as applying AxoDen to the maximum projection image (new Fig 8C).

Use of AxoDen in confocal images obtained at higher magnifications.

We also tested AxoDen in 16-bit images acquired with a classical confocal microscope using the 40x objective (Fig. 8B). We obtained a z-stack of 75 µm in thickness with steps of 5 µm of the prefrontal cortex. The norepinephrine transporter of axons of the Locus Coeruleus (LC) was labeled with a green fluorescent protein. We obtained the Maximum projection of the Z-stack and fed it to AxoDen, which processed the image as intended. Then, we compared the results when either the maximum projection or each image of the Z-stack was fed to AxoDen (Fig. 8C). To analyze the Z-stacked 3D image, each 2D image was converted into gray scale and then binarized using what we called the Global threshold, which was calculated with all the images in the Z-stack. To compare the differences between the images of the Maximum projection and the Z-stack, the 3D stacks were flattened. To flatten the grayscale and binarized Z-stacks, the maximum value in the Z axis of each XY coordinate was selected. To compare the Maximum projection with the Flat Z-stack, we generated Difference images by subtracting the Flat Z-stack from the Maximum projection. The Difference of grayscale images was close to null for the background, while negative values indicate that signal pixels are brighter in the Z-stack than in the Maximum projection. The Difference of binarized images revealed minimal discrepancies, with some scattered pixels showing values of -1, indicating that the Flat Z-stack contained slightly more signal pixels than the binarized MIP. The quantification of signal for the Z-stack was performed in both the 3D stack and the flattened binarized Z-stack (Flat Z-stack, Fig. 8C). The percentages of innervation of the Maximum projection and the Flat Z-stack were very similar, but much higher to that of the 3D Z-stack. This difference is expected, as the 3D z-stack includes a larger volume of background (black pixels), resulting in a lower overall percentage of signal pixels. These findings underscore the importance of consistency in the type of images used as input for AxoDen. Our results indicate that processing the Maximum projection image provides reliable quantification comparable to processing the entire z-stack, without substantial loss of information. Additionally, using individual thresholds for each slice did not significantly alter the results compared to using a global threshold (Fig. 8C, Individual thresholds bar).

In summary, these findings demonstrate that: 1) AxoDen can be effectively applied to 16-bit images acquired with a 40x objective on a confocal microscope; and 2) Processing 2D Maximum projection images is sufficient for accurate axonal quantification, offering the advantage of reduced computational demands compared to processing 3D z-stacks.

3) It is inappropriate to use different thresholds for different images of the same series taken under the same condition. The Otsu method should work well for ROIs with many axons, but probably not for ROIs without axon signals. Once the threshold is determined (for the entire image set or for representative images with many axons), the same threshold must be applied to the entire image set for binarization.

The heterogenicity of innervation of adjacent brain regions that cannot be imaged separately and the inherent variability in the expression of fluorescence (see our response to major point #1 and new Figure 4) require different thresholds for proper pixel classification. Indeed, a threshold that provides good segmentation for a highly innervated ROI will not generate good segmentation for a ROI with low innervation. This does not mean, however, that a ROI with low innervation contains no information. Therefore, a different threshold is needed.

Minor 4) I don't think this procedure is new. Only the GUI is different.

We have been unable to find a paper using segmentation to separate axon from background with the aim to quantify axonal density in a brain area masked with a free form. If we have missed it, we would be grateful the reviewer provides the reference.

5) I don't think it is common to measure ROIs with a rectangular shape. With ImageJ/Fiji, we can easily draw ROIs in any shape and quantify both area and mean intensity. It is unfair to use results from different shapes of ROIs to compare AxoDen vs. Mean Intensity.

While in ImageJ/FIJI one can do free-hand shape ROIs we did not find many examples of other labs doing in the published literature, whereas rectangular ROIs have been used to study axons in these example articles and many others: • Nicolas, C., Ju, A., Wu, Y. et al. Linking emotional valence and anxiety in a mouse insula-amygdala circuit. Nat Commun 14, 5073 (2023). https://doi.org/10.1038/s41467-023-40517-1 • Beyeler A, Namburi P, Glober GF, Simonnet C, Calhoon GG, Conyers GF, Luck R, Wildes CP, Tye KM. Divergent Routing of Positive and Negative Information from the Amygdala during Memory Retrieval. Neuron. 2016 Apr 20;90(2):348-361. doi: 10.1016/j.neuron.2016.03.004. 2016 Mar 31. PMID: 27041499 • Powell JM, Plummer NW, Scappini EL, Tucker CJ, Jensen P. DEFiNE: A Method for Enhancement and Quantification of Fluorescently Labeled Axons. Front Neuroanat. 2019 Jan 11;12:117. doi: 10.3389/fnana.2018.00117. PMID: 30687025 Mean Intensity and AxoDen are fundamentally different independently of the shape of the ROI. While Mean Intensity averages the pixel values, AxoDen segments the pixels into signal and no signal and does not compute the mean of fluorescence intensity.

6) The authors can't draw conclusions with n=2 comparisons. This paper also lacks statistical analyses.

We believe this comment is in reference to the previous Figure 1B showing the n=2 independent Experimenters using AxoDen vs. the square ROI mean intensity approach. Since this one panel is part of larger set of method validation steps, we showed this plot simply to demonstrate the labor time-commitment for a researcher to use AxoDen versus another method. Indeed, the time difference was ~1.6 times faster for each undergraduate researcher, and we provide this information as a descriptive illustration of potential time saved for users. The remainder of the data panels in Figure 1 provides further quantification of AxoDen results in a dataset of n=4 animals collected and analyszed seperately by the n=2 Experimenters, as described in the text. Please also see the answer to the second comment of Rev. 1 above.

7) Line 208. This is useless and inappropriate. We can properly define the threshold without this step.

Unfortunately, the wording of this comment is unprofessional and lacks an explanation. Please refer to answers of points #1 and #3 to address this comment.

8) Line 290. It is more common to take images at 12-bit or 16-bit for quantitative image analysis.

This is the case in purely anatomical studies. For those studies where an anatomical evaluation is complementary to the research project, 8-bit images can be taken. In any case, the number of bits of the image does not affect the performance of AxoDen. This has been addressed in the text in "Considerations for proper use of AxoDen.".

Every digital image is a mosaic of pixels, each encoding information about color and intensity. Commonly, cameras produce color images by synthesizing three primary colors-referred to as channels: Red, Green, and Blue (RGB). Hence, an image inherently possesses three dimensions: 1) width (x-axis), 2) height (y-axis), and 3) the color channels (z-axis). This structure allows us to conceptualize an image as a three-dimensional matrix, wherein each pixel is represented by a trio of values that correspond to the intensity levels of Red, Green, and Blue. The linear combination of the intensity values of the various channels give rise to the wide spectrum of colors we observe in an image. Typically, images are captured in an 8-bit format, meaning the pixel intensity in each channel can assume 28, or 256, possible values ranging from 0 to 255 in steps of 1. In this format, a value of 0 across all channels results in black, while a value of 255 across all channels manifests as white. In images acquired at larger resolution (i.e. 16-bit), the image software will likely show the pixel range between 0 and 255. However, given the increased resolution, the steps between values are smaller than 1.

9) Line 314. This is not so smart. We can use the ImageJ ROI manager to avoid this problem.

Again, this comment is unprofessional, vague and not addressable. Additionally, it is unclear to which problem the reviewer is referring to here.

10) Scale bars are missing in most images.

Scale bars have been added in all images.

11) There is no information about the AAV vectors used in the study.

This information was in the second paragraph of the Methods section under the "Viral vectors" subsection, which included the full description of the construct (serotype, promoter, transgene, enhancer elements, etc.), as well as the titers, the names/location of the vector core that produced each AAV, and when available, the exact product Number for each commercially available AAV.

12) The graphs in Figure 5 and 6 are missing units. It is unclear how the intensity was normalized.

The intensity was normalized to the maximum value. This has been clarified in the legend of the figures.

Reviewer 2 The manuscript is a concise and clear description of a new and useful method. Although we cannot actually test the performance of AxoDen here, so the true evaluation will be left to the User, this reviewer understood the relevance of the manuscript to be delivered to the User.

Thank you for your time and evaluation. If the reviewer wishes to test AxoDen, we have made the everything available to the public prior to the original submission via the web application and the downloadable, stand-alone GUI. We have also provided the test images that can be downloaded from the GitHub for replicating data in the manuscript. The source code is available for review in the GitHub repository. The web address of each can be found in the Methods Section "Code Accessibility" and here: • https://github.com/raqueladaia/AxoDen • https://axoden.streamlit.app/ On the other hand, the manuscript emphasized the advantages from the User's point of view, so there was little description of the technical advantages as the creator, and I would have liked to know a litle more about this in some detail. I would have liked to have received a technical explanation of the part where AxoDen contributed to the improvement compared to the conventional method, even if it was only a short sentence.

We have added the subsection "How AxoDen contributes to the improvement of axonal quantification" (line 618) in the Discussion section to address this comment.

How AxoDen contributes to the improvement of axonal quantification.

The challenge of axonal innervation analysis lies on the large differences in axonal density of adjacent brain regions that are unavoidably imaged together (see Figure 4 and compare CL with MD - see anatomy of CL and MD in Figure 1A). If interested in the information both brain regions, the use of AxoDen allows the researcher to image CL and MD at once, saving time and preserving the fluorophore quality. In such case, to prevent overexposure of CL projections, acquisition intensities must remain low, which results in MD being underexposed. Because AxoDen does not quantify fluorescence intensity and only segments the image into "signal" and "non signal", after masking CL and MD separately (example of CM masking in Figure 1A), the researcher can modify the pixel range for the underexposed brain region to increase the brightness without causing large alterations in the final quantification (Figure 5). This post-acquisition adjustment increases the dynamic range of the masked and cropped MD allowing AxoDen to find a threshold for segmentation, subsequently binarize the image and proceed with quantification. Furthermore, because the masked and cropped brain ROIs are saved as TIFF files and AxoDen provides control figures for each ROI where each step of the algorithm is plotted (example in Figure 2D), the researcher, and anyone interested, can always confirm that no overexposure or extreme modification of the image has been performed.

Could you also describe the amount of future possibilities to detect and technically exclude limitation No.1, and briefly describe the technical reasons for limitation No.2 (optimisation for ×20 lens)? What about the possibility of creating a generalised method that can be used for other objective lenses? Regarding the first limitation, the open-access of AxoDen source code makes AxoDen very easily to combine with other algorithms dedicated to remove artifacts. With regards to the second limitation, AxoDen works because axons can be segmented, and therefore any objective lens that provides images with enough high resolution to distinguish axons will generate images that AxoDen can process. We have added the new section "Use of AxoDen in confocal images obtained at higher magnifications" in which an image at 40x has been fed to AxoDen. For objective lenses of lower magnifications (eg. 4x), axons cannot be visualized and therefore the quantification term "axonal density" is not accurate. This last point has been added in the "Limitations" subsection of the Discussion and tested with a 40x objective lens to show how AxoDen can be generalized as long as axons can be identified.

Use of AxoDen in confocal images obtained at higher magnifications.

We also tested AxoDen in 16-bit images acquired with a classical confocal microscope using the 40x objective (Fig. 8B). We obtained a z-stack of 75 µm in thickness with steps of 5 µm of the prefrontal cortex. The norepinephrine transporter of axons of the Locus Coeruleus (LC) was labeled with a green fluorescent protein. We obtained the Maximum projection of the Z-stack and fed it to AxoDen, which processed the image as intended. Then, we compared the results when either the maximum projection or each image of the Z-stack was fed to AxoDen (Fig. 8C). To analyze the Z-stacked 3D image, each 2D image was converted into gray scale and then binarized using what we called the Global threshold, which was calculated with all the images in the Z-stack. To compare the differences between the images of the Maximum projection and the Z-stack, the 3D stacks were flattened. To flatten the grayscale and binarized Z-stacks, the maximum value in the Z axis of each XY coordinate was selected. To compare the Maximum projection with the Flat Z-stack, we generated Difference images by subtracting the Flat Z-stack from the Maximum projection. The Difference of grayscale images was close to null for the background, while negative values indicate that signal pixels are brighter in the Z-stack than in the Maximum projection. The Difference of binarized images revealed minimal discrepancies, with some scattered pixels showing values of -1, indicating that the Flat Z-stack contained slightly more signal pixels than the binarized MIP. The quantification of signal for the Z-stack was performed in both the 3D stack and the flattened binarized Z-stack (Flat Z-stack, Fig. 8C). The percentages of innervation of the Maximum projection and the Flat Z-stack were very similar, but much higher to that of the 3D Z-stack. This difference is expected, as the 3D z-stack includes a larger volume of background (black pixels), resulting in a lower overall percentage of signal pixels. These findings underscore the importance of consistency in the type of images used as input for AxoDen. Our results indicate that processing the Maximum projection image provides reliable quantification comparable to processing the entire z-stack, without substantial loss of information. Additionally, using individual thresholds for each slice did not significantly alter the results compared to using a global threshold (Fig. 8C, Individual thresholds bar).

In addition, the z-stack of the image acquisition (Keyence BZ-X810, in the mansusript the name was wrong, line 141) in this experiment seems to be a software approximation and not equivalent to the confocal optical section in exact sense, is that correct? If so, is AxoDen compatible for use with confocal optical sections? Furthermore, is it compatible with images acquired by traditional optical microscopy tracing methods such as Biocytin with DAB staining? Thank you for spotting the error on line 141, we have corrected it.

The Keyence BZ-X810 series, as well as traditional confocal microscopes, use a focused laser beam for optical sectioning. Thus, AxoDen is compatible with images obtained from traditional confocal microscopes. We have successfully tested AxoDen with images obtained from a confocal microscope in "Use of AxoDen in confocal images obtained at higher magnifications" and Figure 8 (see response and figure above).

Thank you for bringing up the non-fluorescent staining of Biocytin and DAB staining. With non-fluorescent markers, the imaging step can generate images where the background is white, and the tracing is dark. If we want to use AxoDen for this type of staining, we need to conform with AxoDen requirements. This means that we need to invert the colors of the images so that the tracing is white, and the background is black. We really appreciate and value the point the reviewer has raised and, therefore, we have added the results section "Use of AxoDen in non-fluorescent stainings" and Figure 8A (see figure above).

Use of AxoDen in non-fluorescent stainings.

Traditionally, optical microscopy tracing has relied on non-fluorescent tracers such as the biotin derivative, biocytin, to label axons. Imaging of biocytin staining generates images where unstained tissue (or background) is white and stained tissue (or signal) is dark, and therefore pixels can be easily classified. To test AxoDen on non-fluorescent stainings, we used an image of a coronal brain section containing the intralaminar thalamic nuclei (ILN) from (Fillinger et al., 2018), in which mice were injected with Phaseolus vulgaris leucoagglutinin (PHA-L) in ACC and revealed with 3.3′-diaminobenzidine tetrahydrochloride (DAB) to study the efferents of this brain region (Fig. 8A). First, colors were inverted in ImageJ (Edit > Invert), then, the result was saved as a TIFF file and fed to AxoDen. AxoDen classified signal from non-signal regions, quantifying the percentage of the image containing axons.

Minor points (1) Cities and nations for many companies are not described.

We have added this information for the custom-made viruses. Companies are common biotechnology companies, and thus the reagent identifier is the main information needed to identify the reagent. (2) Several anatomical abbreviations, although common, are used without definition (lines 96, 97, BLA, CM) or before definition (ACC, line 76), which needs corrections.

This has been addressed. (3) The name of the university or laboratory to which the author supposedly belongs was given. lines 64, 144, and github, etc. were also listed, and all are shown.

We are aware of this fact, and we deemed necessary to share the GitHub repository for the reviewers to test AxoDen themselves and to evaluate the source code if wished. We disclosed this in the letter to the reviewer and requested a single blind review process. (4) Regarding the comparison of time and variability, how about stating "n" in the text? lines 175, 178.

This information has been added to the text.
